# Systematic errors in detecting biased agonism: Analysis of current methods and development of a new model-free approach

**DOI:** 10.1038/srep44247

**Published:** 2017-03-14

**Authors:** H. Ongun Onaran, Caterina Ambrosio, Özlem Uğur, Erzsebet Madaras Koncz, Maria Cristina Grò, Vanessa Vezzi, Sudarshan Rajagopal, Tommaso Costa

**Affiliations:** 1 Ankara University, Faculty of Medicine, Department of Pharmacology, Molecular biology and Technology development unit, Sıhhiye, Ankara, Turkey; 2 Istituto Superiore di Sanitá, Dip. Farmaco, Rome, Italy; 3 Departments of Medicine and Biochemistry, Duke University Medical Center, Durham, North Carolina, USA

## Abstract

Discovering biased agonists requires a method that can reliably distinguish the bias in signalling due to unbalanced activation of diverse transduction proteins from that of differential amplification inherent to the system being studied, which invariably results from the non-linear nature of biological signalling networks and their measurement. We have systematically compared the performance of seven methods of bias diagnostics, all of which are based on the analysis of concentration-response curves of ligands according to classical receptor theory. We computed bias factors for a number of β-adrenergic agonists by comparing BRET assays of receptor-transducer interactions with Gs, Gi and arrestin. Using the same ligands, we also compared responses at signalling steps originated from the same receptor-transducer interaction, among which no biased efficacy is theoretically possible. In either case, we found a high level of false positive results and a general lack of correlation among methods. Altogether this analysis shows that all tested methods, including some of the most widely used in the literature, fail to distinguish true ligand bias from “system bias” with confidence. We also propose two novel semi quantitative methods of bias diagnostics that appear to be more robust and reliable than currently available strategies.

The term biased agonism indicates the situation in which an agonist can have large differences of efficacy in promoting receptor interactions with different transduction proteins. It primarily concerns the family of G protein coupled receptors (GPCRs), as these ligand-activated proteins must bind additional transduction proteins to trigger diverse and sometimes conflicting signalling pathways^1^. Consequently, an agonist with efficacy biased towards favouring a particular transducer interaction may cause a disproportional stimulation of the corresponding signalling pathway, thus effectively “biasing” the pattern of receptor responsiveness towards a more restricted and specific biological function. The functional selectivity (or agonist-directed signal trafficking)^2^,^3^,^4^ that results from the activation of a receptor with a biased agonist may have important therapeutic implications, such as leading to the discovery of new drugs with reduced side effects and improved risk-benefit index^5^,^6^,^7^,^8^,^9^,^10^. This perspective explains the surge of interest in biased signalling across the current biochemical and pharmacological literature.

However, measuring ligand bias is not trivial. In fact, disproportional responses to agonists also occur when there is no “true” efficacy bias. For instance, there can be differences in signal when comparing upstream with downstream responses of a pathway that starts from the same receptor-transducer interaction. The reason is that the strength of ligand responses observed in most signalling pathways does not vary in direct proportion to the extent of receptor/transducer activation triggered by agonist binding. Moreover, the analytical methods used to determine biological signalling usually add further nonlinearity to the input-output relationship between ligand-induced activation and signal. Collectively, the term system bias is used to represent such additional mechanisms that generate apparent bias in signalling. System bias is either an artefact of analytical methods or the result of amplification in signalling network chains, which varies from one cell type to another. Thus, system bias makes it difficult to predict the actual signalling selectivity that may occur *in vivo* or allow for optimization of bias by modifications of a ligand’s structure. In contrast, ligand bias depends on the different efficacies of an agonist for distinct receptor-transducer complex formation. It is thus encoded in the chemical identity of the ligand.

Many methods have been proposed for discriminating ligand bias from system bias and quantifying the extent of biased efficacy in agonists^11^,^12^,^13^,^14^. The majority of such methods rely on the definition of ligand’s efficacy given in classical receptor theory^15^,^16^. Despite the inherent oversimplification of this theoretical framework, those strategies appear to work, at least when tested over an extensive set of computer simulated data^17^. However, exhaustive experimental verification of their validity is still lacking. Two previous studies comparing the relative abilities of several computing methods in detecting biased efficacy^14^,^18^ revealed significant divergences in the ability to identify biased ligands. Such results cast a legitimate doubt on whether the abundant phenomenology of biased signalling described in the literature really represents cases of agonists that have biased efficacy.

A major problem concerning the accuracy of ligand bias computations is “circular proving”. Most of currently known biased agonists were identified using the presently available methods; thus, there is no panel of independently proven biased ligands that can be used for checking the accuracy of current methods. In this study we reversed the question by checking the ability of each method to correctly identify balanced, i.e. unbiased, agonism. We expect that an accurate method of bias diagnostics should find no bias when there is none. To assess this, we compared different ligand-induced responses that stem from the same receptor-transducer interaction, in which no biased efficacy is possible. We analysed the most commonly used strategies of bias analysis, including unpublished variants inspired from the same theory (7 methods altogether). As shown here, these methods produce a high rate of statistically significant false positive results, thus lacking the experimental rigor and robustness that would be necessary for a compound screening program, even at the earliest stages. We also present two new semi-quantitative strategies of bias diagnostics that are both more reliable and robust than previous methods.

## Results

### Gs, Gi or arrestin bias in β_2_AR

We studied 11 β_2_AR agonists with varying efficacy for coupling the receptor to Gs, Gi or β-arrestin. Receptor-transducer coupling was measured by BRET (Bioluminescence Resonance Energy Transfer) assays (see Methods). We used membranes from Gs-KO cells to assess β_2_AR-Gi interactions. In this preparation, the BRET signal originating from the rLuc-β_2_AR donor and Gβ-rGFP acceptor is abolished by PTX treatment, indicating mediation by Gα_i/o_-subunits (Supplementary Figure S1). Thus, by comparing ligand-induced BRET responses in membranes from Gs-KO cells and Gs-reconstituted cells treated with pertussis toxin (PTX), we could evaluate the differential ability of agonists to promote receptor coupling to Gi and to Gs, respectively.

**Figure 1:**
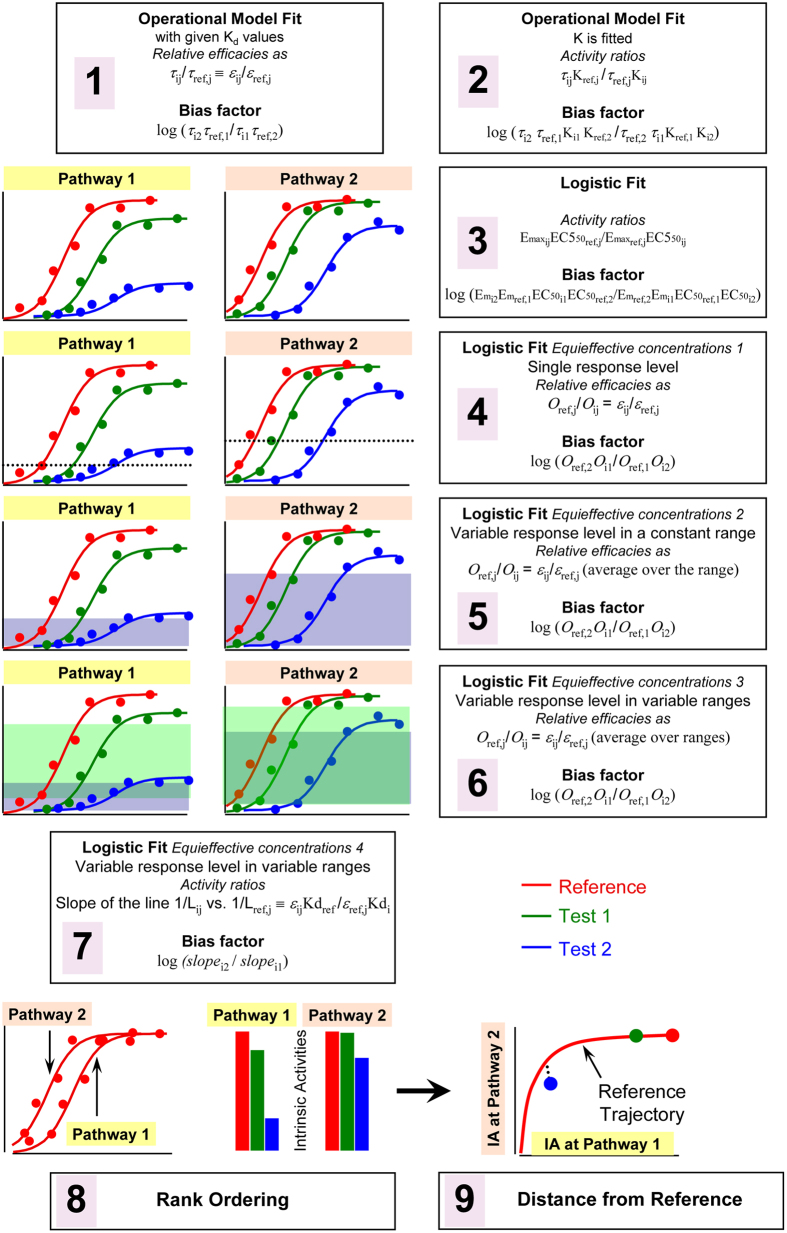
Schematics of the bias diagnostic methods used in the study. Methods are numbered from 1 to 9. The information obtained using these methods is schematically shown for a comparison of two different bioassays using 1 reference and 2 test agonists, as indicated in the graphs. The basic principles and the output of the methods are given in the numbered boxes. The first two methods are based on the fitting of the operational model, whereas methods 3 to 7 use fitting of a 3-parameter logistic equation to the CR curves. Methods 8 and 9 do not require concentration-response curve information from test agonists. Methods 4–7 are all based on the null theory (see Methods section for details). Methods 1, 4, 5 and 6 require experimental determination of unconditional affinities, (unlike methods 2, 3, 7, 8, 9). A more detailed description of methods 8, 9 is in Fig. 2.

**Figure 2:**
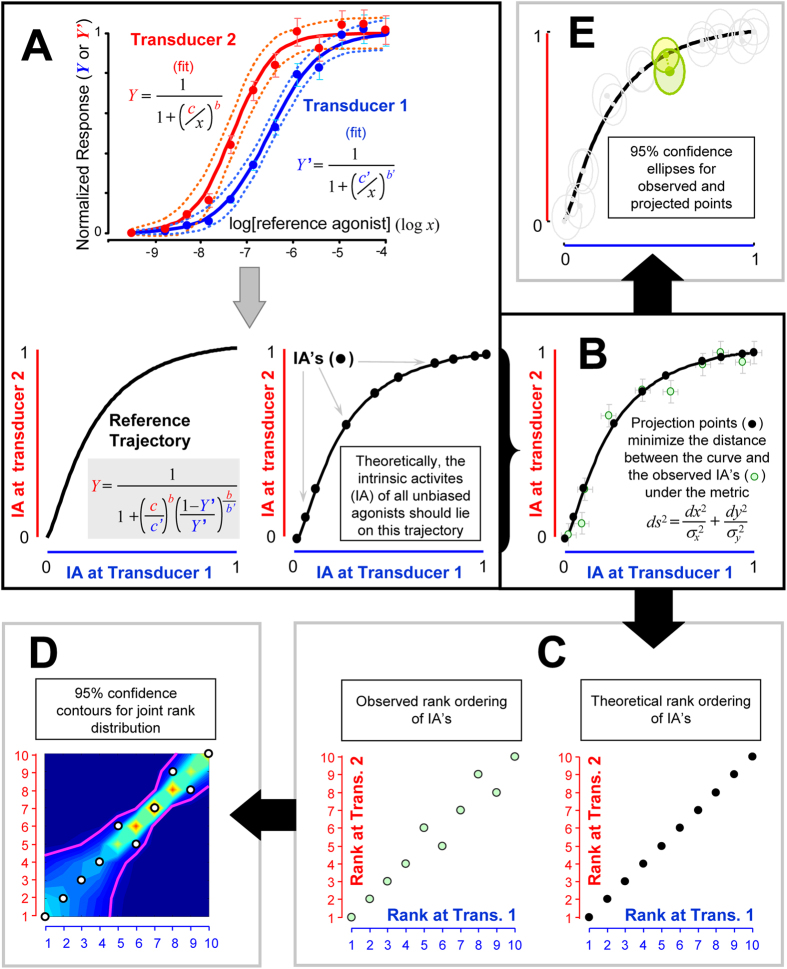
Simplified descriptions of two new model-independent methods of bias diagnostics based on intrinsic activities (IA’s) data. Schematic illustration of the approach using simulated CR curves of a reference full agonist and the intrinsic activities of 10 (unbiased) test agonists in two different transducer systems that are coupled to the same receptor. Test agonists with varying efficacies (but identical for the two transducers), and different transducers are simulated by two different transfer functions. (**A**) CR curves of the full reference agonist at two different responses define a trajectory in the plane of normalized responses. In theory, the IA’s of unbiased test agonists lie on this trajectory. (**B**) Observed IA’s (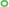) deviate from the trajectory due to random experimental noise. Under the null hypothesis that the test agonists are unbiased, the locations of the corresponding IA’s on the trajectory (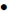) can be estimated by minimizing the distance between the observed IA’s and the trajectory using the indicated metric, in which the displacements in the two axes are weighted with the inverse variance of the experimental error. (**C**) The projected points on the trajectory (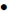) can be translated into a linear rank ordering. Experimental noise disturbs this rank ordering (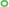). (**D**) Exact sampling distribution and the joint 95% confidence contour of the random disturbance are computed by Monte Carlo simulations (see text for details). (**E**) Significant deviations from the reference trajectory can also be directly evaluated by comparing the 95% confidence ellipses of the observed IA’s and corresponding projected points on the trajectory. As an example, two points (i.e. observed and projected) and their corresponding confidence ellipses are coloured in the picture to emphasize intersecting ellipses that indicate insignificant deviation (i.e. no bias).

**Figure 3:**
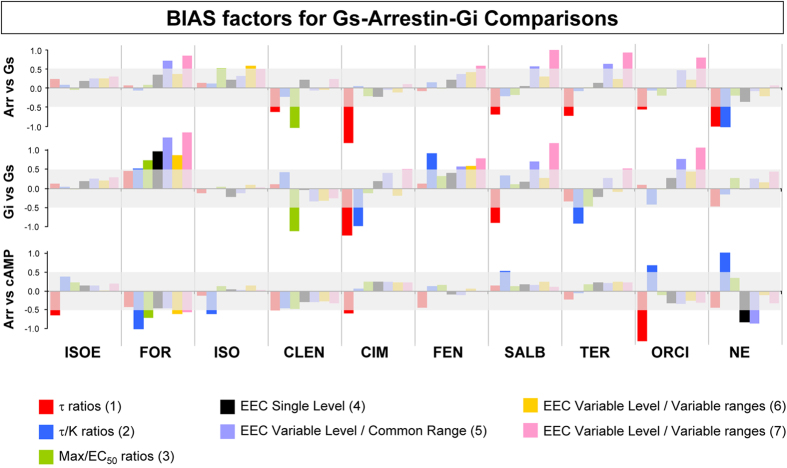
Estimated bias factors of adrenergic agonists using different methods. For the indicated set of agonists, bias factors are calculated using the indicated methods (numbered as in Fig. 1). The relevant comparisons are marked on the bias-factor axes. Agonist-induced β_2_AR-β-arrestin 2 (Arr), β_2_AR-Gs and β_2_AR-Gi interactions were directly measured using BRET assays in 2B2 cells. Agonist-induced cAMP responses are measured in HEK-293 cells stably expressing Glo-Sensor probe. The relevant CR curves are shown in Supplementary Figures S2–S5. The transparent grey bands in the pictures indicate 95% confidence limits for the bias factors as determined by Monte Carlo simulations (see Supplementary Method for details). Abbreviations are: ISOE, isoetharine; FOR, formoterol; ISO, isoproterenol; CLEN, clenbuterol; CIM, cimaterol; FEN, fenoterol; SALB, salbutamol; TER, terbutaline; ORCI, orciprenaline; NE, norepinephrine; EEC, equi-effective concentrations. Epinephrine is used as the reference agonist in all comparisons.

From concentration-response (CR) curves of the ligands for Gs, β-arrestin and Gi interactions bias factors were calculated with 7 different methods using Epinephrine as reference agonist (see Methods and Figs 1 and 2 for the description of the bias assessment strategies) and are summarized in Fig. 3. A common 95% confidence band of ±0.5 about zero bias, as assessed by a Monte Carlo strategy (see details in Supplementary Methods), indicates the boundary of statistical indeterminacy.

The bias factors quantifying efficacy differences for the transducer pairs [β-arrestin vs. Gs] and [Gi vs. Gs] are shown in the first two panels of Fig. 3. Some ligands show a significant bias towards transducers other than Gs, (e.g. CLEN and CIM, apparently biased towards arrestin and Gi). For most of the agonists, however, it is evident that the different methods generate uncorrelated bias assessments. One exception is formoterol (Fig. 3, middle panel), which shows significant Gs vs. Gi bias according to most of the methods used (discussed below). To resolve this apparent lack of consistency in the analysis, we performed a confirmative test. We measured the CR curves of the same set of agonists in intracellular cAMP accumulation, which may be considered an alternative indicator of ligand-induced receptor-Gs interaction, and calculated the bias factors between β-arrestin and cAMP responses (Fig. 3, bottom). Theoretically, these additional bias factors should agree with those obtained for β-arrestin vs. Gs (Fig. 3, top), if the methods can detect the same quantity (i.e. true biased efficacy between arrestin and Gs). Thus, for the given ligands, a strong positive correlation among the bias factors in the top and bottom panels should be expected. On the contrary, the calculated correlation coefficients (with method-number in parentheses) were remarkably weak, or even negative in one case: 0.06(1), −0.50(2), 0.33(3), 0.23(4), 0.24(5), 0.04(6), 0.00(7). These results indicate that the bias factors shown in Fig. 3, even when statistically significant, cannot be attributed to the presence of true ligand bias with any confidence.

### Apparent biased agonism in negative control experiments

To further analyse this inconsistency, we examined a number of negative controls. A good negative control experiment consists in evaluating pairs of responses that unquestionably result from the same receptor-transducer interaction. Any bias detected in such cases represents “non-statistical noise” (i.e. a statistically significant false positive result), because the methods are applied to a “bias-impossible” trial by definition. Thus, the imbalance between responses is the result of system bias, which all the computing methods examined in this study should be able to eliminate.

**Figure 4:**
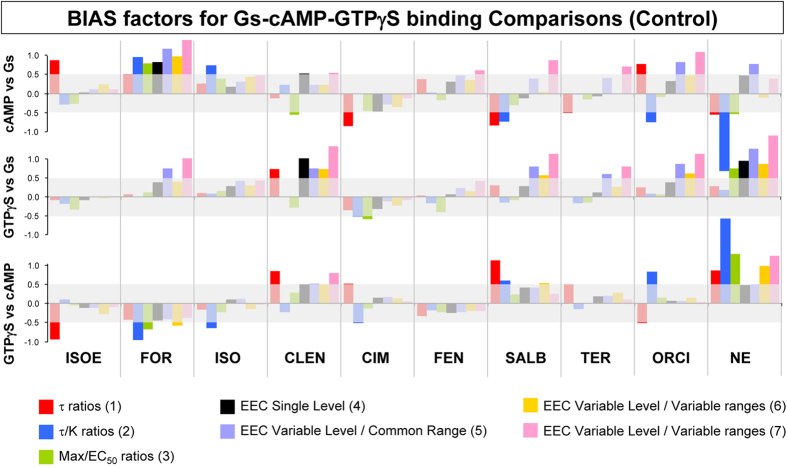
Bias factors of adrenergic agonists in three bioassay comparisons reporting β_2_AR-Gs interaction. Bias factors for the indicated set of agonists are calculated with the indicated methods. (numbered as in Fig. 1). The different comparisons are indicated on the corresponding bias-factor axes. Agonist-induced β_2_AR-Gs interaction was measured in three different ways: (1) Receptor-Gs interaction obtained in BRET assay (indicated as Gs in the picture), (2) ^35^S-GTPγS binding, and (3) cAMP accumulation using the Glo-Sensor probe. See Supplementary Figures S2, S5, and S6 for CR curves that were used in these analyses. All three determinations are expected to carry the same information of efficacy about the tested agonists, as all bioassay gauge receptor interaction with the same transducer. The transparent grey bands in the pictures indicate 95% confidence limits for the bias factors as determined by Monte Carlo simulations (see Supplementary Method for details). Abbreviations are: ISOE, isoetharine; FOR, formoterol; ISO, isoproterenol; CLEN, clenbuterol; CIM, cimaterol; FEN, fenoterol; SALB, salbutamol; TER, terbutaline; ORCI, orciprenaline; NE, norepinephrine; EEC, equi-effective concentrations. Epinephrine is used as the reference agonist in all comparisons.

Negative controls consisted of 3 equivalent measures of receptor-Gs interactions. Ligand-induced β_2_AR-Gs coupling was assessed by: (1) BRET, (2) enhancement of GTPγS binding in β_2_AR-Gs fusion protein, and (3) increase of intracellular cAMP accumulation. The first two assays detect the interaction at the most proximal level of receptor stimulation, and both depend on the same molecular event, namely GDP-release from Gαs, determined in isolated membranes. The third assay depends on Gs-mediated activation of adenylyl cyclase in intact cells. The results of these experiments are shown in Fig. 4. The general variation pattern of bias factors determined in these negative controls is comparable to that observed previously (Fig. 3). There is an overall similarity between the “bias-impossible” test bench of Fig. 4 and the biologically-relevant comparisons of Fig. 3 with regards to the propensity of the 7 methods to generate inconsistent and uncorrelated ligand bias factors. This suggests that none of the results obtained with these procedures can be confidently attributed to the existence of true biased efficacy in a ligand.

In principle, when comparing a cell dependent response with one obtained in membranes, a false bias factor may result if some ligands have a strong bias towards arrestin: the enhanced interaction with arrestin and consequent internalization could diminish the expected cAMP response compared to the Gs interaction obtained in membrane, where no internalization occurs. However, if such a perturbation existed, it should be consistent with the β-arrestin bias of the ligand that was already determined (Fig. 3). Yet, the significant bias factors (for some ligands with some method) that were detected in cAMP response versus membrane-dependent BRET/GTPγS responses did not appear to be related to a β-arrestin interference. For example, NE, ORCI and CIM, showing a significant β-arrestin bias with at least one method in the previous analysis (Fig. 3), might be expected to exhibit a bias towards the membrane assay response, due to the quenching effect of β-arrestin-mediated internalization on cAMP. In contrast, these ligands showed either a bias in the opposite direction (NE) or gave inconsistent results (Fig. 4).

To examine convergence, we first evaluated the within-method correlations of the bias factors in Fig. 4. Theoretically, the bias factors calculated from cAMP vs. Gs (BRET) comparisons (Fig. 4, upper panel) should be highly correlated with those calculated from GTPγS-binding vs. Gs (BRET) comparisons (Fig. 4. middle panel), because the information delivered is reporting on the same biochemical event. However, the correlation coefficients calculated for all ligands were remarkably low: 0.01(1), −0.24(2), 0.18(3), 0.70(4), 0.72(5), 0.17(6), 0.57(7).

We then evaluated inter-method correlations by pooling bias factors from Figs 3 and 4 (correlation matrix in Table 1). Apart from relatively high correlations among methods 4–7, which are minor variations of the same procedure (Fig. 1), the overall correlations among the methods were weak. Such poor correlations do not seem to be related to inaccurate determination of specific model parameters. For example, the results from the subset of methods that do not rely on independently measured K_d_ values (i.e. methods 2, 3, 7) were not intrinsically more correlated than the others. In summary, the seven methods documented here, although equivalent in theoretical background, give uncorrelated results when challenged on experimental data. This means that they cannot consistently measure moderate levels of biased efficacy that may exist in the studied ligands.

As a final challenge for consistency, we examined an additional set of negative controls. For this assay the same response (cAMP accumulation) from the same test-panel of agonists was examined in cells with differing levels of β_2_AR or Gs expression using two different analytical means of cAMP detection (GloSensor vs. radioimmunoassay [RIA]). This analysis can directly assess the ability of the methods to remove different kinds of system bias. In fact, the differences in receptor or transducer expression can generate a “biological” type of system bias (i.e., differing sensitivity of the signalling pathway to the range of ligand efficacy). Conversely, different methods of cAMP detection can generate “analytical” system bias, because biosensor signals, unlike absolute determinations via RIA, tend to saturate rapidly at high cAMP concentration^19^. Consistency in the results implies that all methods should report a lack of ligand bias, as there cannot be difference of efficacy across assays that measure the same transduction pathway. However, we observed significant bias factors in this analysis (Supplementary Figure S8). In fact, most of the ligands exhibited at least one positive hit in the comparisons. Moreover, unlike in previous analyses, the bias factors assigned to a given ligand were often concordant across the 7 methods. Surprisingly, some of the bias factors, which should be zero for these negative controls were the largest of the study, with log ratios occasionally approaching 2.

Altogether, these analyses provide compelling evidence that there is a major discrepancy between experimental reality and the theoretical background on which ligand bias computing methods are based.

### Bias diagnostics based on the analysis of relative intrinsic activities of ligands

The lack of reliability documented above might reflect excessive sensitivity of bias computing methods to minor deviations between agonist CR curves and model predictions. With this hypothesis in mind, we sought to develop a strategy of ligand bias diagnostics that breaks the model-dependency of currently available methods. We reasoned that intrinsic activity (IA, i.e., the relative effects of ligands at receptor saturation) represents a model-free and robust indicator of the power of agonist to activate a receptor system. In fact, the ranking of IA in agonists provides direct information on the relative efficacy of ligands (set aside cases of extreme signal amplification, where virtually all agonists may display identical levels of maximal response). Accordingly, biased efficacy can be detected as a change in IA rank ordering across two different transduction systems. We developed two alternative methods to assess such a change. Both rely on the analysis of agonists IA’s with respect to a reference trajectory computed from the difference in the CR curves of the full agonist obtained in two different assay responses. (Methods, Fig. 1, panels 8, 9 and Fig. 2).

**Figure 5:**
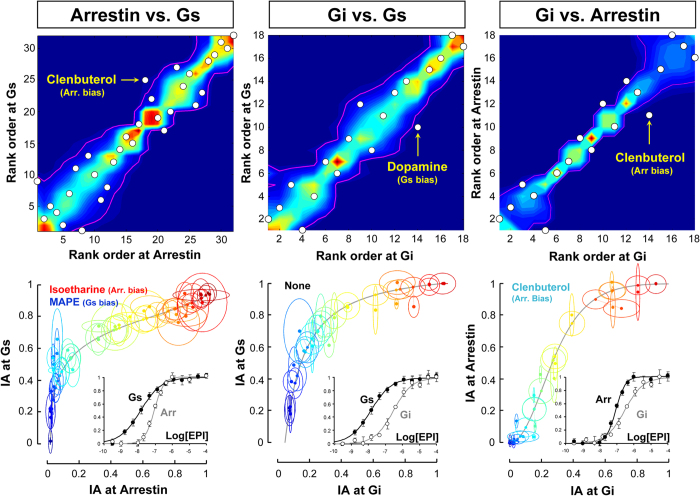
Bias analysis of β_2_AR interaction with three transduction proteins using two novel model-free methods. Upper and lower panels shows the results of rank ordering and distance methods, respectively, at indicated comparisons (columns). Insets in the lower panel are the reference CR curves for epinephrine at indicated responses. *Upper Panel*: Simulated joint densities of ranks are shown in a rainbow scale, and their confidence contours are indicated as magenta curves. Observed ranks for agonists’ intrinsic activities are indicated as white circles. *Lower Panel*: The solid grey curves are reference trajectories for epinephrine at indicated responses. Observed and projected intrinsic activities (solid circles), and their 95% confidence ellipses are coded with the same colour for a given agonist. Different agonists are shown with different colours. See text for detailed description of the methods. All responses are measured by using BRET assays in 2B2 cells as explained in the text. Agonists that are determined to be biased in each method and comparison are indicated in the corresponding panels. Norepinephrine was found to be marginally biased in all comparisons in the upper panel (white circles lying on the x-axis), but the determination of its maximum response was problematic due its low potency. The numbers of test agonists evaluated in arrestin-Gs, Gi-Gs and Gi-arrestin comparisons, are 32, 18 and 18, respectively. See Supplementary Figure S7 for the raw data and the list of ligands that are evaluated in each response.

Maximal ligand-induced receptor coupling to Gs, Gi or β-arrestin 2 (assessed by BRET assays using a large set of test agonists) are compared in Fig. 5. The intrinsic activities of the majority of ligands lie within the joint dispersion of the expected rank order (upper panels) and do not significantly depart from the corresponding reference trajectories (lower panels). Only a few ligands displayed a statistically significant biased efficacy in at least one of these comparisons. However, these positive identifications were not consistent across the two variants of the method (compare method 8 with 9, on top and bottom panels of Fig. 5), which indicates that possible biased efficacies possessed by such compounds must be of marginal significance.

**Figure 6:**
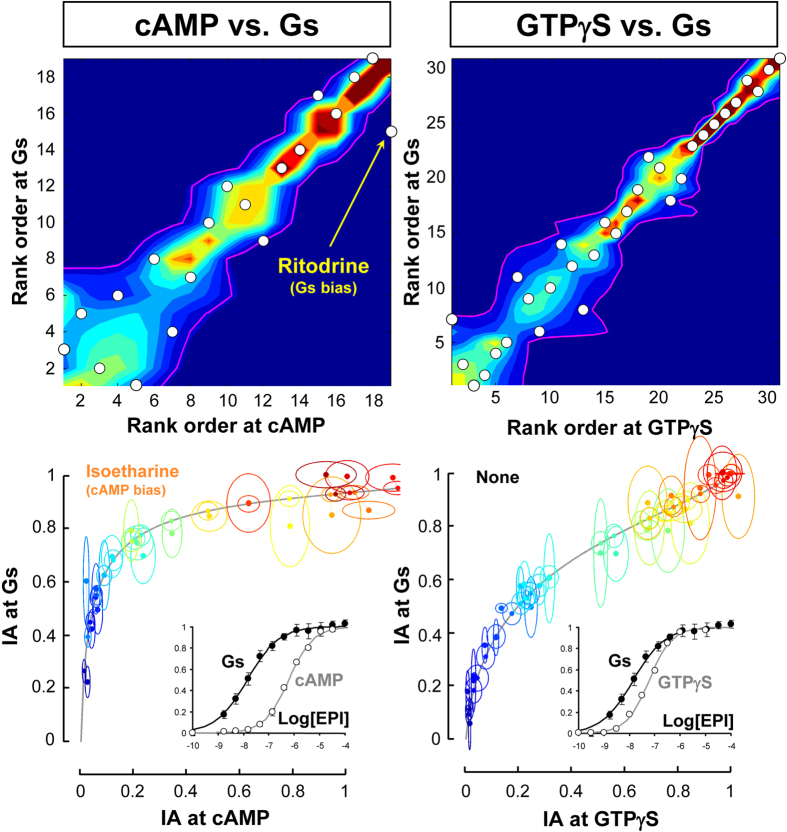
Performance of the intrinsic activity-based methods in negative control assay comparisons. Upper and lower panels shows the results of rank ordering and distance methods, respectively, at indicated comparisons (columns). Insets in the lower panel are the reference CR curves for epinephrine at indicated responses. *Upper Panel*: Simulated joint densities of ranks are shown in a rainbow scale, and their confidence contours are indicated as magenta curves. Observed ranks for agonists’ intrinsic activities are indicated as white circles. *Lower Panel*: The solid grey curves are reference trajectories for epinephrine at indicated responses. Observed and projected intrinsic activities (solid circles), and their 95% confidence ellipses are coded with the same colour for a given agonist. Different agonists are shown with different colours (see text for detailed description of the methods). Receptor-Gs interaction is assessed by a BRET assay in 2B2 cell membranes, cAMP accumulation is measured in Glo-Sensor expressing HEK-293 cells, and GTPγS binding is determined by radioligand binding assay in HEK-293 cell membranes, as explained in the text. Agonists that are determined to be biased in each method and comparison are indicated in the corresponding panels. The numbers of test agonists evaluated in cAMP-Gs and GTPγS-Gs comparisons are 19 and 31, respectively. See Supplementary Figure S7 for the raw data and the list of ligands that were evaluated in each response.

The propensity of the new methods to generate false positive bias results was checked using negative controls, as described for the other methods. The intrinsic activities of ligands measured in three different assays of the receptor-Gs interaction (cAMP accumulation, GTPγS binding and BRET measurements) were compared (Fig. 6) in both method 8 (upper panel) and 9 (lower panel). Both results were consistent with an overall lack of biased efficacy in the ligands. Only two agonists, isoetharine and ritodrine, yielded a potential bias in the cAMP-Gs comparison (left panels), but not consistently in both methods. In the GTPγS vs. BRET comparison (right panels, Fig. 6) none of the 31 test ligands displayed a significant bias. Thus, the overall rate of positive hits in negative control experiments obtained with the new methods is considerably lower than the rate observed in the other seven methods analyzed before.

**Figure 7:**
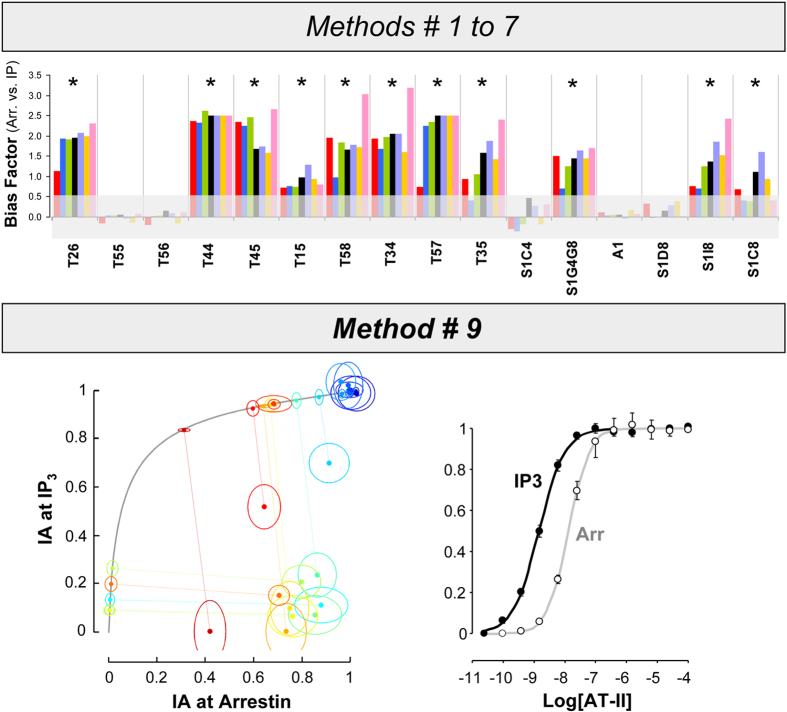
Bias analyses of angiotensin 1 receptor agonists using CR-curve or intrinsic activity (IA) data. Sixteen test agonists at AT1R are evaluated for their bias agonism in arrestin recruitment and G_q_-mediated inositol phosphate (IP) production responses by using the seven methods based on the CR-data, and the model-independent “distance” method (i.e. method #9) based on the IA’s, as indicated in the picture (see text for detailed description of the methods). Angiotensin II is used as the reference agonist in all analyses. Results are presented in the same format as in Figs 3, 4 (methods 1 to 7) or as in Figs 5, 6, lower panels (method 9). Ligands showing significant bias factors are indicated by asterisks in the upper panel of the picture. Eleven, out of 16 test ligands that are consistently found to be arrestin-biased by the seven methods, are also diagnosed to be significantly biased by the distance method; i.e. the 11 points that represent the same 11 ligands above, are found to be located under the reference trajectory (solid grey curve in the lower-left panel). The latter points have non-overlapping confidence ellipses with their corresponding trajectory points.

To address the question of whether this enhanced reliability is achieved at the cost of reduced detection power, we analyzed a positive control consisting of angiotensin receptor 1 agonists previously shown to exhibit a strong level of biased efficacy for arrestin^14^,^20^. As shown in Fig. 7 (upper panel), the peptides were first tested using the 7 model-based methods, all of which yielded a clear and statistically significant arrestin bias. Although the reliability of such strategies is significantly improved in the case of strongly biased ligands, there were large differences (up to one order of magnitude) in the extent of bias estimated for the same ligand by different methods. This indicates that the ability of such computations to converge into an exact quantification of the difference in efficacy, while true in principle, is only an approximation in practice. We next analysed the same data with our newly developed model-free approach. For the comparison of IA’s in arrestin recruitment and Gq-mediated IP production only method 9 can be used, since the majority of the ligands in the test panel are biased, making rank ordering (i.e. method 8) unfeasible. As shown in Fig. 7 (bottom panel) the 11 agonists exhibited highly significant deviations from the reference trajectory consistent with strong efficacy bias towards arrestin, and confirm that the method is fully competent in detecting the presence of biased efficacy in ligands.

### Overall rating of bias diagnostic methods

To evaluate the relative performance of the bias computing methods examined in this study we focused on two indicators. One is the residual mean squares (RMS) deviation from the no-bias base level. The other is the average number of ligands with a significant biased efficacy found by each method. Unlike the former, the latter can be applied to both model-dependent (1–7) and model-independent (8, 9) methods, thus allowing a global comparison. RMS deviations for methods 1–7 were estimated separately for the comparisons involving diverse transducers (where biased efficacy may be potentially present, i.e. the Gs-Arrestin-Gi data from Fig. 3) and the comparisons involving a single transducer (where no biased efficacy can exist, e.g. the single transducer assays of Fig. 4). A global estimate pooling both cases was also calculated.

**Figure 8:**
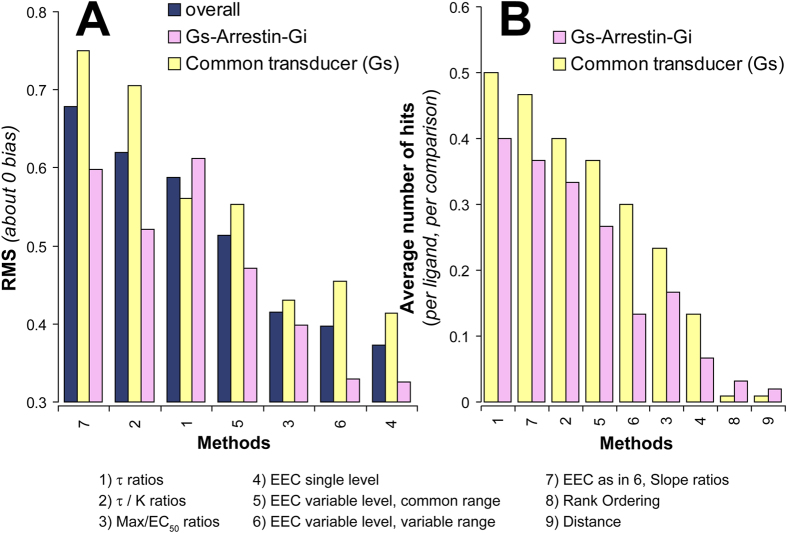
Global comparisons of the different methods of biased agonism analysis. The overall rates of bias diagnostics of the different methods are assessed. (**A**) Average departures from zero-bias are given as root mean square (RMS) deviations for indicated methods. The bias factors given in Figs 3 and 4 are used for the analysis. Bias factors that correspond to receptor-Gs, -arrestin and -Gi interactions are evaluated separately from those that correspond to receptor-Gs interaction, cAMP accumulation and GTPγS binding to Gs (as indicated in the picture). By definition, the latter set is expected to be free from any true bias, as all the evaluated responses result from a common interaction of receptor with Gs. The results of the analysis of the pooled data are also given (indicated as “overall” in the picture). (**B**) Average number of biased compounds determined by the indicated methods are shown. As in panel A, comparisons involving a common transducer (i.e. Gs) are evaluated separately from those that involve multiple transducers (i.e. Gs, Gi and arrestin). The data set given in Figs 3 and 4 (for methods 1–7), and the data set given in Figs 5 and 6 (for methods 8 and 9) are used for the analyses. Average number of significant bias (hits) is given as the total number of hits divided by the total number of agonists used in the analysis and the number of comparisons in which these agonists are involved. Note that in all cases (except in method 8 and 9) more bias is diagnosed in “bias irrelevant” comparisons (i.e. those that involve a common transducer in the compared responses) than in “bias possible” comparisons.

As shown in Fig. 8A, the 7 model-dependent methods vary in the propensity of attributing false bias factors to ligands, which oddly seems to be even greater in “bias-impossible” than in “bias-possible” cases. Nonetheless, the general similarity of the trends observed in the two cases enforces our conclusion that none of the bias factors detected by these methods correspond to a true efficacy difference of the ligand for the transduction proteins under study.

The global method comparison based on the frequency of positive hits allows the generation of a common “reliability scale” (Fig. 8B). It is worth noting that two strategies based on the operational model (methods 1 and 2), and the one based on slope ratios of reciprocal equi-effective concentrations (method 7) are most prone to generate false positive results. In contrast, method 4, i.e., ratios of the equi-effective occupancy ratios at a single response level, lies at the opposite extreme. It is also clear from this comparison that the new model-free methods of bias diagnostics introduced in this study (methods 8 and 9) generate the lowest level of statistically significant false ligand bias.

## Discussion

This study was originally designed to measure differences of efficacy among adrenergic agonists for inducing β_2_AR interactions with their main transduction proteins, Gs, Gi and β-arrestin 2. Taking advantage of the ability of Mg^2+^ ions to enhance weak receptor-Gi/o protein interactions and using *Gnas*-KO cell lines^21^ reconstituted or not with the Gα_s_ gene, we established a simple but efficient BRET assay for comparing receptor-Gs and receptor-Gi coupling in membranes. However, as our computations of biased efficacy used a large number of methods, serious inconsistencies in data analysis became evident. We thus turned our attention on two different objectives: a full-fledged reliability testing of the currently available methods and an attempt to develop more reliable strategies for diagnostics and quantification of biased agonism.

We have tested 7 different methods of ligand bias computations. All of them are subsumed in the main strategy of quantitative pharmacology for extracting information about ligand intrinsic efficacy from bioassays. The first group (i.e. methods 1−3 in Fig. 1, where # 2 is the most widely used in the literature^13^) is based on transduction ratios estimated by the operational model^22^. The second is a mechanism-free group of approaches (i.e., methods 4−7 in Fig. 1) based on the null method, which cancels system-dependent inter-cellular differences by taking the ratio of agonist concentrations that give equal response; here we explored all possible implementations of this second approach, some of which were not described in detail before, thus representing new alternatives to the more frequently used methods. Despite the differences, all 7 methods are borne in the same traditional theory of receptor action. Specifically, the idea that properties encoded in the chemical structure of the agonist, such as receptor recognition and response-evoking power (i.e., affinity and efficacy), can be discriminated from system parameters, such as cellular concentrations of receptor or transducer and the sensitivity of the signalling network to the extent of transducer activation. Given this common framework, all methods should yield convergent and correlated results in the identification of biased agonism, except for divergences resulting from variations of sensitivity or the different ways in which numerical and experimental errors cross-interact in determining the final result. A categorically different bias-diagnostics method suggested by Barak and Peterson^23^ was excluded from the present analyses, since it is not designed to extract efficacy information from bioassay data and thus distinguish system-bias from ligand-bias.

As shown here, these methods cannot identify with confidence whether ligands are weakly or not biased. Two indicators that clearly demonstrate the unreliability of bias factors are the lack of correlation among the test results and the high tendency to generate false positive scores of biased agonism in bioassay comparisons in which only system bias was present (i.e., negative controls). It should be noted that it is possible that different reporters of the same signalling pathway, e.g., G protein recruitment, GTPγS binding and cAMP for G protein signalling, may be differentially activated by different ligands depending on the mechanisms that underlie their activation^24^.

The first is documented by the weak or even negative correlations among the bias factors computed within and between methods. Part of this may be due to the fact that these bias factors were essentially fitting noise from unbiased signalling, thus resulting in a lack of correlation between those values. However, this outcome is in contrast with the expectation that the methods should measure, albeit with different precision or sensitivity, the same quantity. The second indicator, i.e., the high frequency of false positive bias assignments, shows that the methods often fail to accurately discriminate system bias from true ligand bias. This inaccuracy is even more severe if we consider that the Monte Carlo-based limit of statistical significance used here is far more conservative than the commonly used tests based on error propagation rules^12^ (e.g., see Supplemental Figure S1E).

It is worth discussing what major factor might underlie the propensity of the methods to generate erratic and false-positive results. Arguably, the limitations of these assays are similar to those previously discussed for the traditional model of agonist action^22^. Specifically, the lack of chemical identity for the ‘intrinsic efficacy’ parameter (*ε*) and the pragmatic but abstract nature of the stimulus-effect relation limit both conceptual value and experimental verification of the main assumptions on which the classical theory is based. Thus, the idea that bioassay analysis can dissect molecular parameters of receptor-transducer interaction from agonist-independent components of physiological response might be fundamentally flawed.

However, as shown previously^17^, there is a simple mathematical relationship between ε as defined by Furchgott^15^ and the macroscopic cooperativity that best describes in energetic units the allosteric effect of the agonist in stabilizing a transducer-bound receptor complex^25^,^26^. Although serendipitous, this relationship predicts that, within a fairly wide level of agonism, the determination of relative efficacies for multiple ligands through such methods should asses the size of that allosteric effect, even if only as a relative value. Moreover, we do not find a tight relationship between the extent of model dependency in a method and its propensity to show false positive results. In fact, methods 1 and 2 that require estimating 3 mechanistic parameters of the operational model (τ, K_A_ and *r*_max_) generate the same level of statistically significant noise as method 7, which only relies on two minimal assumptions: i.e., that equal stimuli produce equal effects and that the stimulus is the product of ε and the fraction of bound receptors. The above considerations suggest that neither parameter definitions nor the dependence from model formulation might be the culprit for the unreliable outcome of the methods under test.

Interestingly, the method with the lowest score of false positive results (Fig. 5A) is the one using the fewest amounts of data points derived from the agonists CR curves. In fact, method 4 is identical to other null method-based strategies (5−7), except that the ratio of equi-effective agonist concentrations is computed using only two crossing points at a single response level. Thus, we suspect that the performance of the method is worse the greater density of information is extracted from the agonists CR curves. Conceivably, in the presence of system bias even minor anomalies that do not significantly alter the “goodness-of-fit” in each individual curve might still be sufficient to give rise to nonzero bias factors in this type of analysis.

The main assumption in classical receptor theory is that functional responses are compared under steady-state conditions of the molecular events generating signalling. Thus, slow kinetics is one factor that might distort CR curves. Agonists with very slow response-eliciting dynamics exist and this anomalous behaviour can affect some bioassays far more than others. As experimentally shown recently^27^, positive bias factors erroneously indicating differences in transducer efficacy can entirely result from abnormal kinetics. Many additional potential sources of error, such as nonlinear interactions among pathways that are not codified in the theory, including competition among transducers for the same receptor or feedback control mechanisms acting crosswise among parallel signaling cascades, may cause the anomalies described here; but a comprehensive discussion goes far beyond the scope of this report. We may assert conclusively that the bias factors calculated with these methods should be treated with extreme caution.

To mitigate the dependence of bias analysis from theoretical models and CR curves analysis, we developed a strategy that is designed to detect changes in rank orders of intrinsic activity between signalling pathways arising from different transducers. The underlying idea is that the difference between the CR curves of a full agonist in two bioassays can subsume the variations of intrinsic activities in all other agonists with lesser efficacy. From this difference, the expected relationship between rank-orders of all ligands in the two assays can be used as guide for detecting deviations due to biased efficacy and assess statistical significance. We conceived two equivalent methods based on this approach, which differ on whether statistical testing is applied to a re-mapped rank ordering graph of the data or to the distance between observed points and predicted trajectory.

As shown in the results, these new methods are far less prone to generate false positive results in negative control assays, but maintain full capability to identify biased agonists. Although they still depend on CR curve analysis, this is limited to one reference agonist. For all the other test ligands intrinsic activity is the only needed information. Such type of information is far less costly and time-consuming to generate than that requiring full CR curves, which is an advantage, particularly in high throughput screening efforts involving a very large number of substances.

However, these new methods also come with limitations. One is that their optimal diagnostic power is achieved when comparing “low-amplification” bioassays, in which the information about efficacy is more present in the intrinsic activity than in the EC_50_ of the ligands. Such test systems may not always be available. Moreover, method 8 requires a reasonably large number of unbiased ligands in the test-panel, in order to maintain statistical resolution. More importantly, although the new methods may provide a measure of deviation from the unbiased trajectory-line for rating biased agonism, they cannot quantify the extent of difference in efficacy. We do believe, however, that giving up the ability to quantify a theoretical parameter for an increase in reliability is still a convenient trade off, particularly if the results of bioassays are intended to be used for expensive screening campaigns in drug discovery or for deciding which ligands should be co-crystallized with receptors in X-ray structural analysis.

## Methods

### Experimental procedures

#### Reagents and Drugs

Cell culture media, reagents, and foetal bovine serum (FBS) were from Invitrogen, Gibco or Biological Industries; restriction enzymes were from New England Biolabs. Coelenterazine, bisdeoxycoelenterazine (coelenterazine 400a) and luciferin (Na salt) were from Biotium Inc. or Nanolight Technology. Radiolabeled [^35^S]-GTPγS and [^125^I]-iodocyanopindolol were from Amersham. Adrenergic ligands were from Tocris, Bachem, Santa Cruz or Sigma-Aldrich. Pertussis toxin was from List Biologicals or Sigma-Aldrich. All other reagents were from Sigma-Aldrich, Merck or Fisher Scientific.

#### Plasmids

Retroviral expression vectors encoding human β_2_-AR receptors tagged at the C-terminal with rLuc and Gβ_1_-subunit or β-arrestin 2 tagged at the N-terminal with rGFP were prepared as described previously^28^. Plasmid encoding the luciferase-based intracellular cAMP probe GloSensor 22 F was purchased from Promega. The retroviral vectors with various antibiotic resistance genes (the pQC series), the plasmid encoding the envelop protein VSV-G and the packaging cells GP2-293 were purchased from Clontech.

#### Cell cultures and transfections

HEK-293 (Human Embryonic Kidney) cells were grown in DMEM supplemented with penicillin (100 u/ml), streptomycin (100μg/ml) and 10% FBS in a humidified atmosphere of 5% CO_2_ at 37 °C. The 2B2 *Gnas*^E2-/E2-^ fibroblasts (kindly provided by Murat Baştepe, Harvard Medical School, Boston, MA, USA), where the 2^nd^ exon of the *Gnas* gene was ablated, were grown in the same conditions except that DMEM:F12 (1:1) medium supplemented with 5% FBS was used. 2B2 cell clones that stably express rLuc-tagged β_2_-AR alone or together with rGFP-tagged Gβ_1_, β-arrestin 2 or rGFP-tagged Gβ_1_+G_αsL_ were obtained by retroviral transfection followed by appropriate antibiotics selections; G418 (500 μg/ml), hygromicin (100 μg/ml) and/or puromycin (5 μg/ml) whenever appropriate. Native or β_2_-AR-over expressing HEK-293 cells, and 2B2 cells (stably co-transfected with β_2_-AR+G_αsL_) were stably transfected with GloSensor 22 F by using lipofectamine 2000 reagent (Thermo Fisher Scientific) as instructed by the supplier.

#### Membrane preparations

Cells were detached by PBS-EDTA, pelleted at 200 × g for 5 min at room temperature, resuspended in homogenization buffer (5 mM Tris-HCl pH 7.4, protease inhibitor mixture, Roche Diagnostics), and homogenized by passing the suspension 10–15 times through a 26 G syringe tip on ice. The homogenate was centrifuged at 450 × g for 10 min at 4 °C, and the resulting supernatant at 100,000 × g for 30 min at 4 °C (Beckman Coulter Optima LE-80K). The pellet was suspended in 50 mM Tris-HCl (pH 7.4), 10 mM MgCl_2_, protease inhibitor mixture, and 25% sucrose at a protein concentration of approximately 2 mg/ml, and stored at −80 °C. Membranes that were used in BRET assays were prepared and stored exactly in the same way except that only distilled water + protease inhibitor mixture was used in all steps.

#### BRET assays for β2-AR- Gs,- Gi or -arrestin interactions

BRET signals were recorded and analyzed essentially as described previously^29^,^30^. Briefly, receptor-Gβ_1_ interactions, which require the presence of a Gα interaction, were measured in 96-well white plastic plates (Packard Opti-plate or Greiner) using membrane preparations in a total volume of 100 μl of PBS. Assays were started by pipetting coelenterazine to the plate wells containing membranes in PBS, 10 mM MgCl_2_. The incubation lasted 8 minutes (which is the time necessary to attain a stable basal BRET ratio that remains constant for about 20 min). Next, ligands at varying concentrations were rapidly added using multi-channel pipettes and incubated for additional 3 minutes before counting the plate. The intensity of luminescence in the wells was counted sequentially at two wavelengths using either a Victor Light (equipped with two bandpass filters, 450/10 nm and 510/10 nm) or an Envision luminometer (bandpass filters, 470/30 and 515/30 nm) (both from Perkin Elmer). Kinetic measurements were used to verify that the change of BRET ratio induced by ligands was at plateau with 3 minutes of incubation. Two lines of 2B2 cells co-expressing the rLuc-β_2_-AR and rGFP-Gβ_1_ constructs, one without and the other with retrovirally-expressed G_αsL_, were used to distinguish β2-AR interactions with Gi/o and Gs. In membranes prepared from the first cell line the BRET signal is Gi-dependent. In membranes prepared from the Gs-reconstituted line previously treated with pertussis toxin (10 ng/ml, 18 h) the BRET signal is Gs dependent (see also Supplementary Figure S1). Experiments were conducted independently in two different laboratories. Depending on the specific activities of the membrane preparations, 1–3 μg of membrane protein per well, and 1–5 μM (final concentration) of coelenterazine were used. All these experiments were pooled and the average results are presented. Receptor-β-arrestin 2 interactions were measured in intact cell monolayers using the luciferase substrate analogue bisdeoxycoelenterazine (bDOC, 5 μM) as described previously^28^. Basal-subtracted concentration-response (CR) curves of BRET ratios (510 nm/450 nm or 515 nm/470 nm) for different agonists were normalized with respect to the epinephrine maximal response.

#### cAMP Measurements

GloSensor-22F expressing cells were seeded in 96-well white plates at a density of 25 × 10^3^ cells/well 24 hours before the experiment. Two hours before the assay, cells were washed once with PBS, and further incubated 105 minutes in PBS + 25 mM glucose in the presence of 3 mM luciferin in a total volume of 60 μl. The assay was started by adding agonists at varying concentrations and 1 mM (final) IBMX (3-isobutyl-1-methylxanthine) on the thermostatic plate of the luminometer set at 37 °C in a total volume of 100 μl/well. Luminescence form each well was then counted every 30 s with 0.5 s integration time for 20 minutes. Ligands and IBMX were diluted in PBS + 25 mM glucose. cAMP response was determined as peak of luminescence intensity, area under the 20 min luminescence-time curves, or the initial rate of luminescence increase. All three methods gave similar results. Resulting CR curves for each ligand were corrected either with respect to the forskolin (10μM) response obtained in parallel experiments, or with respected to the maximal responses measured at saturating concentrations of the ligands. All curves were then further normalized with respect to epinephrine’s maximum response. The same procedure was followed for cAMP measurement by RIA, except that the assay was started by adding ligands + IBMX at 37 °C, and terminated after 5 minutes by adding 100 μl 0.2 N HCl. The RIA procedure for determination of cAMP concentration in the cell extracts was described before^31^. Data were first corrected for the number of living cells determined by MTT assay (Sigma-Aldrich) in parallel experiments, and then normalized with respect to the epinephrine responses.

#### Arrestin recruitment and Inositol 1-Phosphate Assay of angiotensin 1 receptor agonists

Data for arrestin recruitment and inositol 1-phosphate signalling are from Rajagopal *et al*.^14^, where full details for those assays are noted.

#### GTPγS binding in β_2_AR-Gas fusion proteins

The preparation of cells expressing β_2_AR-Gα_s_ fusion protein and the [^35^S]GTPγS binding assay used to determine agonists responses have been described previously^32^.

#### Radio-ligand binding assays

Receptor binding experiments were carried out in the membrane preparations from pertussis toxin- treated (200 ng/ml, overnight) 2B2 cells that express rLuc-tagged β_2_-AR. Membranes (0.5–1 μg protein/well) were incubated with [^125^I]-iodocyanopindolol (15000 dpm/well; ~20 pM) in the presence of varying concentrations of competitor ligands, GDP (100μM), AlCl_3_ (20 μM) and NaF (10 mM) in a total volume of 250 μl buffer (100 mM KCl, 10 mM MgCl2, 50 mM Tris-HCl pH 7.4) for 2 hour at 37 °C in 96-well plates. The reaction was stopped by rapid filtration through Whatman GFB glass fiber filters by using a cell harvester (Skatron Instruments). Radioactivity on the filters was counted by using a scintillation counter (Wallac MicroBeta 1450 Trilux). K_d_ values for the competitor ligands were estimated by means of the nonlinear regression of the numerical solution of bi-molecular binding equation that includes nonspecific binding parameters in the presence of multiple ligands, using a visual Basic MS Excel routine. For each ligand, the average of 2 independent binding curves obtained in quadruplicates was used for the regression analysis. In all regressions the K_d_ value for the radioligand was fixed to 40 pM, as determined by stauration binding assays. On average, the receptor density in these membranes were calculated to be 23 ± 3 pmol/mg protein (n = 29). Serial dilutions of salmeterol were made in the presence of 0.1% (w/v) bovine albumin for all experiments.

#### Analysis of the agonist CR curves

The curves obtained in various bioassays were fitted either with the operational model (methods 1, 2) or with a 3-parameter logistic function (methods 4–7). For both models we used the log-form of the original equations, which behave better than the original ones in the fitting procedures. For the operational model, as suggested by Kenakin *et al*.^13^ we used


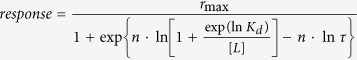


For the 3-parameter logistic, we used:


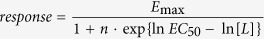


The estimated parameters are *r*_max_, *n* (slope factor), ln*τ*, (and ln*K*_*d*_) in case of the operational model, or *E*_max_, *n* (slope factor) and ln*EC*_50_ in case of the 3-parameter logistic model. Note that the slope factor in the operational model is not equivalent to that of the logistic model. For the estimation of regression summary statistics, including the 95% band for the fitted curves, we used the standard asymptotic methods (see for example Bates and Watts^33^). We used the average of at least 3 independent triplicate or quadruplicate CR curves in each regression analysis. Exact numbers of data points and replicates used in the analyses are indicated case by case in figure legends. All computational procedures were programmed using scripts written in MS Excel Visual Basic and/or Matlab programming language, whenever appropriate.

### Analysis of biased agonism

In the present analyses we used nine different methods of bias computing (indicated below with numbers in the parentheses). The first seven comprise most of the currently used strategies, which can be classified in two groups: those based on the operational model and those based on the null method. Additionally, we introduce here two novel methods that represent a third group of model-independent interrelated strategies of bias diagnostics.

#### Strategies based on the operational model

The operational model^22^ is a special case of Stephenson’s theory of receptor action^16^, which is schematically summarized as follows:

A ligand (*L*) binds to the receptor according to a bimolecular reversible interaction:


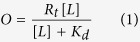


where *O* is receptor occupancy, *R*_*t*_, total receptors, and *K*_*d*_ the equilibrium dissociation constant for the binding reaction. The ligand-receptor complex is assumed to produce a “stimulus” (*s*) in the cell that is proportional to both *O* and the ligand’s intrinsic efficacy *ε,* i.e., *s* = *ε O*.

The observed biological response *r* is related to the stimulus by an unspecified but monotonic function, i.e., response, *r* = *f(s*). If we posit that *f* may be a 3-parameter hyperbolic function, where *r*_max_ is the global maximum, *n* a slope factor, and *K*_E_ a sensitivity parameter, we get the operational model. This is defined as:


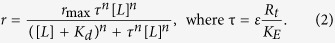


Equation (2) implies that the maximum response of the ligand (*E*_max_) is defined as


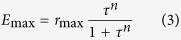


Estimates of *τ*, which contain information about ligand efficacy, might in principle be obtained by fitting the above equation to agonist CR curves. The ratios of *τ* values with respect to a reference agonist in a given response yield the relative ε values of the agonists, as the system dependent parameters, *R*_*t*_, and *K*_*E*_, cancel in the *τ* ratios. Thus, for any given ligand the log-ratio of relative efficacies at two different responses provides the bias factor^34^,^35^.

However, due to the identifiability problem in the regression of the operational model, *K*_*d*_ and *τ* cannot be estimated independently especially for strong agonists. This leaves only two viable options. Either *K*_*d*_ values are experimentally measured and used to constrain the regression, thus obtaining a reliable estimate of *τ* from the computer fit^14^,^36^, or the two parameters are computed as unresolved *τ/K*_*d*_ ratios from the regression^13^. In the latter case no relative efficacy of agonists can be measured from each individual response^17^, but the bias factors are still computable as ratios of *τ/K*_*d*_ ratios across different responses. Selecting the best of these approaches is often not a free choice, because ligand binding constants cannot be determined in all receptor systems. Therefore, we used both strategies here. Specifically:

(1) The regression of the model was constrained using the *K*_*d*_ values of the agonists, which were obtained from radioligand binding experiments in the absence of receptor-transducer interaction; then the bias factors were computed as (log) ratios of relative efficacies (see Supplementary data S9 for the full set of competition binding curves and corresponding log*K*_*d*_ values).

(2) The *K*_*d*_ values were treated as unknown parameters and let free to change in the model regression; then the bias factors were computed as (log) ratios of *τ/K*_*d*_ ratios. (panels 1 and 2, Fig. 1)

(3) We also computed bias factors using a simpler approach, by determining the *E*_max_/*EC*_50_ ratios^37^. In fact, in the operational model the median effective concentration is defined as


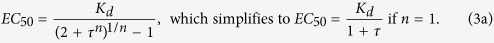


Thus, in case of a slope factor very close to one, the ratio *E*_max_/*EC*_50_ is:


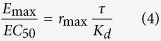


Since *r*_max_ (the maximal possible response in each measured biological system) is a system dependent parameter, thus common to all agonists, it is cancelled when all *E*_max_/*EC*_50_ ratios are scaled to that of a reference ligand. Hence, the (log) ratio of the relative values of *E*_max_/*EC*_50_ at two different responses provides a bias factor estimate. This is equivalent to that obtained from the second strategy described above, if the slope factors are all equal, or very close to unity. We estimated *E*_max_ and *EC*_50_ by fitting a 3-parameter logistic function to the CR curves. (see panels 3 in Fig. 1).

#### Strategies based on the “null method” according to Stephenson-Furchgott theory

The “null hypothesis” in SF theory states that equal stimuli should produce equal responses. Thus, two different ligands, L_r_ and L_t_ (with subscripts indicating reference and test agonist) will generate equal responses when *ε*_*r*_*O*_*r*_ = * ε*_*t*_*O*_*t*_. Therefore, the ratio of occupancies, *O*_*r*_/*O*_*t*_, measured at equi-effective concentrations of the agonists corresponds to the relative efficacy, *ε*_*t*_/*ε*_*r*_. Bias factors can be computed from the (log) ratios of the equi-effective occupancy ratios measured for two different biological responses. There are again two options in applying this strategy, depending on whether *K*_*d*_ values can or cannot be measured experimentally.

In the first case, fractional occupancies at equi-effective concentrations ([*L*]_eq_) are calculated as [*L*]_eq_/([*L*]_eq_ + *K*_*d*_). By inverting the 3-parameter logistic functions fitted to the agonist CR curves, equi-effective concentrations are computed. For derivation of equi-effective occupancy ratios we used three possible variants of the approach:

(4) Selecting a single response level common to all agonists (panel 4, Fig. 1).

(5) Using multiple response levels within a range common to all agonists (panel 5, Fig. 1).

(6) Finally, using multiple response levels within a variable range that was optimized according to each pair of reference/test agonist under study. (panel 6, Fig. 1).

Note that in methods (**5**) and (**6**), all the determinations made at the multiple response levels were averaged to compute occupancy ratios and relative efficacies, as all should converge to a unique theoretical estimate, with variations only due to experimental error.

In the second case (i.e., unknown K_d_’s), SF theory suggests a strategy that does not require equi-effective occupancy ratio calculations, hence, no information about the binding constant K_d_. Briefly, at equi-effective concentrations of reference (*L*_*r*_) and test ligand (*L*_*t*_), the theory predicts the following equality (with *K*_*r*_ and *K*_*t*_ being the respective binding constants of reference and test agonists):


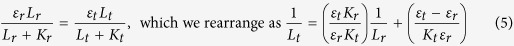


(7) Equation (5) shows that there is a linear relationship between the reciprocal equi-effective concentrations of reference and test agonist, with slope yielding the relative value of *ε/K*_*d*_ of the test agonist with respect to that of the reference agonist. Accordingly, we compute the bias factors from the (log) ratio of the slopes of the double-reciprocal lines determined in the two different assay systems. This procedure is schematized in panel 7, Fig. 1. A very similar approach, albeit used in a different context, has been proposed by Rajagopal *et al*.^14^.

#### Model-independent strategies

We have developed two new semi-quantitative diagnostic tools to assess bias. Both are based on the evaluation of relative intrinsic activities (IA’s) of ligands observed in two signalling pathways (i.e., the maximal responses of all agonists normalized to that of a full agonist producing the highest level of response in both assays). The underlying empirical principle is that the rank order of agonist IA’s for two different responses that are mediated by the same pair of receptor/transducer are invariant when there is no ligand bias. Although system bias may generate strong non linearity in the relationship between sets of IA’s measured at two different responses, it cannot change the relative rank of the agonists in these responses. This rank only depends on relative efficacies. However, in the presence of non linearity combined with experimental error, it can be virtually impossible to assess whether a true and significant change in rank ordering exists.

To solve the problem we took advantage of the idea that the CR curve of a full agonist in each bioassay spans all possible levels of intrinsic activity that all other agonists can show in that system. Thus, from the analysis of the best fitting full agonist CR curves generated in the two assays we can specify the exact shape of the theoretical trajectory that the relationship between IA’s of all unbiased agonists should follow. This “reference trajectory” becomes a ruler for testing if changes in rank ordering of intrinsic activity exist and are statistically significant. Two different strategies were used (schematized in Fig. 1, panel 8 and 9).

##### Analysis of changes in rank ordering (method 8)

This analysis is best suited to the situation in which a larger set of unbiased ligands comprising a wide range of IA’s coexist with a smaller subset of biased agonists. In the absence of agonist bias, all ligands IA’s should lie on the reference trajectory (Fig. 2, panel A). Deviations that are not justified by experimental error identify biased agonists. To perform the analysis, theoretical and experimental points are translated into a uniform rank ordering map. Experimental error perturbs this ordering in a peculiar way, depending on the shape of the reference trajectory and the error structure of experimental data (Fig. 2, panels B and C). Under the statistical null hypothesis (i.e., no agonist bias is present), a joint distribution for the random error perturbations can be built and used to assess the significance of potentially biased ligands. The whole procedure is described by the following sequel of steps:

(a) Construct the reference trajectory from the fitted CR curves of the full agonist in the two signalling pathways as shown in Fig. 2, panel A, by fitting a two-parameter logistic equation onto the normalized CR curves of the reference agonist obtained at two different responses. Using the fitted values of EC_50_’s (i.e. c and c’), slope factors (i.e. b and b’) and the equation shown in Fig. 2, panel A (i.e. Y vs. Y’) draw the reference trajectory on a plot whose coordinates are the ranges of IA for the two bioassays under test.

(b) With the two experimental IA values of each test ligand, generate the observed point and identify the corresponding “theoretical” point (Fig. 2, panel B), i.e., the point on the reference trajectory that minimizes the distance between the curve and the observed point. For distance calculation we used an anisotropic metric that takes into account the statistical uncertainty in both axis (see Supplemental method and figure SM2[A–C] for further details).

(c) Assign to each observed and projected point two integers representing the sequence numbers – i.e. rank orders – in the sorted list of intrinsic activity for each response. The rank orders of the theoretical points trace the identity line, since the reference trajectory is a monotonic curve (Fig. 2C).

(d) Perturb the positions of such theoretical points by adding to their *x* and *y* coordinates two random numbers generated from uncorrelated normally distributed random variables with zero means and standard deviations equal to those of the corresponding experimental points; re-sort the perturbed IA’s and re-assign new rank orders as described above; record the resulting rank orders; repeat this step for 500,000 times.

(e) From the recorded set of rank orders, calculate the joint frequencies of the ranks. Find the envelope that encloses 95% of the joint ranks (i.e., the 95% confidence contour of the joint distribution).

(f) Identify as biased agonists those ligands that lie outside the confidence contour by comparing experimentally determined ranks with the joint distributions of the ranks obtained under the statistical null hypothesis (schematics in Fig. 2D).

##### Analysis of deviations from expected trajectory (method 9)

Unlike the rank ordering analysis described above, this second approach does not require coverage of a wide range of IA in test ligands; thus, it can be applied to a few or even a single test agonist. It consists in testing the statistical significance of the distance between any observed value of the IA plot (i.e. representing the experimentally measured IA’s of a given ligand in the two bioassays) and the corresponding “theoretical” point on the reference trajectory. Both theoretical trajectory and the projections of observed IA’s on this reference trajectory are determined as described in steps *(a)* and *(b)* of the previous section. Next, we compute 95% confidence ellipses for the observed points and corresponding theoretical points on the trajectory (Fig. 2E). The ellipses for the observed points are constructed from the experimentally observed errors of the IA’s; those of the theoretical points are calculated from the asymptotic 95% confidence band of the nonlinear regression of the CR curves (blue or red dotted curves in Fig. 2A; see also Supplemental Figure SM2, panels D-E, for more details). An agonist is considered biased when the observed and the projected trajectory points have non-intersecting confidence ellipses. Excel templates and Matlab scripts that help to implement the above computations are available from the first author.

## Additional Information

**How to cite this article:** Onaran, H. O. *et al*. Systematic errors in detecting biased agonism: Analysis of current methods and development of a new model-free approach.. *Sci. Rep.* **7**, 44247; doi: 10.1038/srep44247 (2017).

**Publisher's note:** Springer Nature remains neutral with regard to jurisdictional claims in published maps and institutional affiliations.

## Supplementary Material

Supplemental Information

## Figures and Tables

**Table 1:** Correlation matrix for bias factors.

Methods	1	2	3	4	5	6	7
**1**	1						
**2**	0.27	1					
**3**	0.35	**0.62**	1				
**4**	**0.58**	0.22	0.45	1			
**5**	0.43	0.07	0.47	**0.86**	1		
**6**	**0.67**	0.50	**0.73**	**0.83**	**0.81**	1	
**7**	0.37	0.21	0.49	**0.77**	**0.93**	**0.83**	1

Each coefficient in the table indicates the correlation between bias factors calculated by using indicated pair of methods for the same set of agonists in all the response comparisons used in the present study. Methods are numbered as in Fig. 1. The set of test and reference agonists and the compared responses are given in Figs 3 and 4.
