# Supplementary material for: Systematic errors in detecting biased agonism: Analysis of current methods and development of a new model-free approach
Source: Sci Rep. 2017 Mar 14;7:44247. doi: 10.1038/srep44247 (PMC5349545; doi:10.1038/srep44247)
Supplement: Supplemental Information [file srep44247-s1.pdf]

Supplementary information to:

Systematic errors in detecting biased agonism. Analysis of current methods and development of a new model-free approach.

H. Ongun Onaran[1*], Caterina Ambrosio[2], Özlem Uğur[1], Erzsebet Madaras Koncz[1], Maria Cristina Grò[2], Vanessa Vezzi[2], Sudarshan Rajagopal[3], Tommaso Costa[2*]

[1]Ankara University, Faculty of Medicine, Dept. of Pharmacology, Molecular biology and Technology development unit, Sıhhiye, Ankara, Turkey.
[2]Istituto Superiore di Sanitá, Dip. Farmaco, Rome, Italy
[3]Departments of Medicine and Biochemistry, Duke University Medical Center, Durham, North Carolina, USA.

Supplementary Data

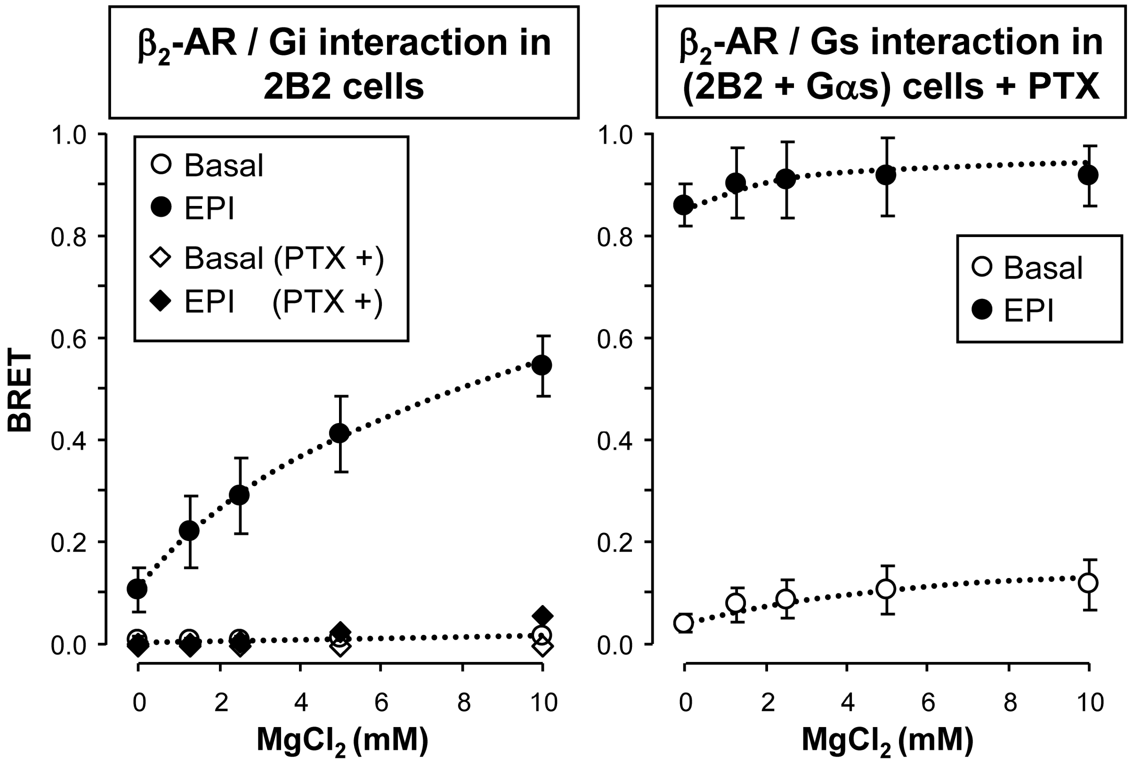

Figure S1. Mg-dependent interaction of β2-AR with Gi in 2B2 cells assessed by BRET. In membranes from 2B2 cells lacking $G_{\alpha s}$ and expressing rLuc-β_2AR+rGFP-Gβ_1 (indicated as 2B2 cells, left panel), the EPI-induced BRET signal is PTX-sensitive and it is stongly enhanced at higher Mg concentrations. In contrast, in membranes from 2B2 cells reconstituted with $G_{\alpha sL}$ by retroviral transduction the epinephrine response is PTX-insensitive and only slightly affected by Mg concentrations (right panel). Thus, to asses ligands ability to couple receptor to Gi/o or to Gs individually, BRET assays were conducted in the presence of 10 mM $MgCl_2$ and we used membranes from 2B2 cells for measuring the first interaction and membranes from 2B2 cells reconstituted with Gs and treated with PTX for the second interaction. Data are means of three or four independent experiments. Error bars indicate standard error of the mean.

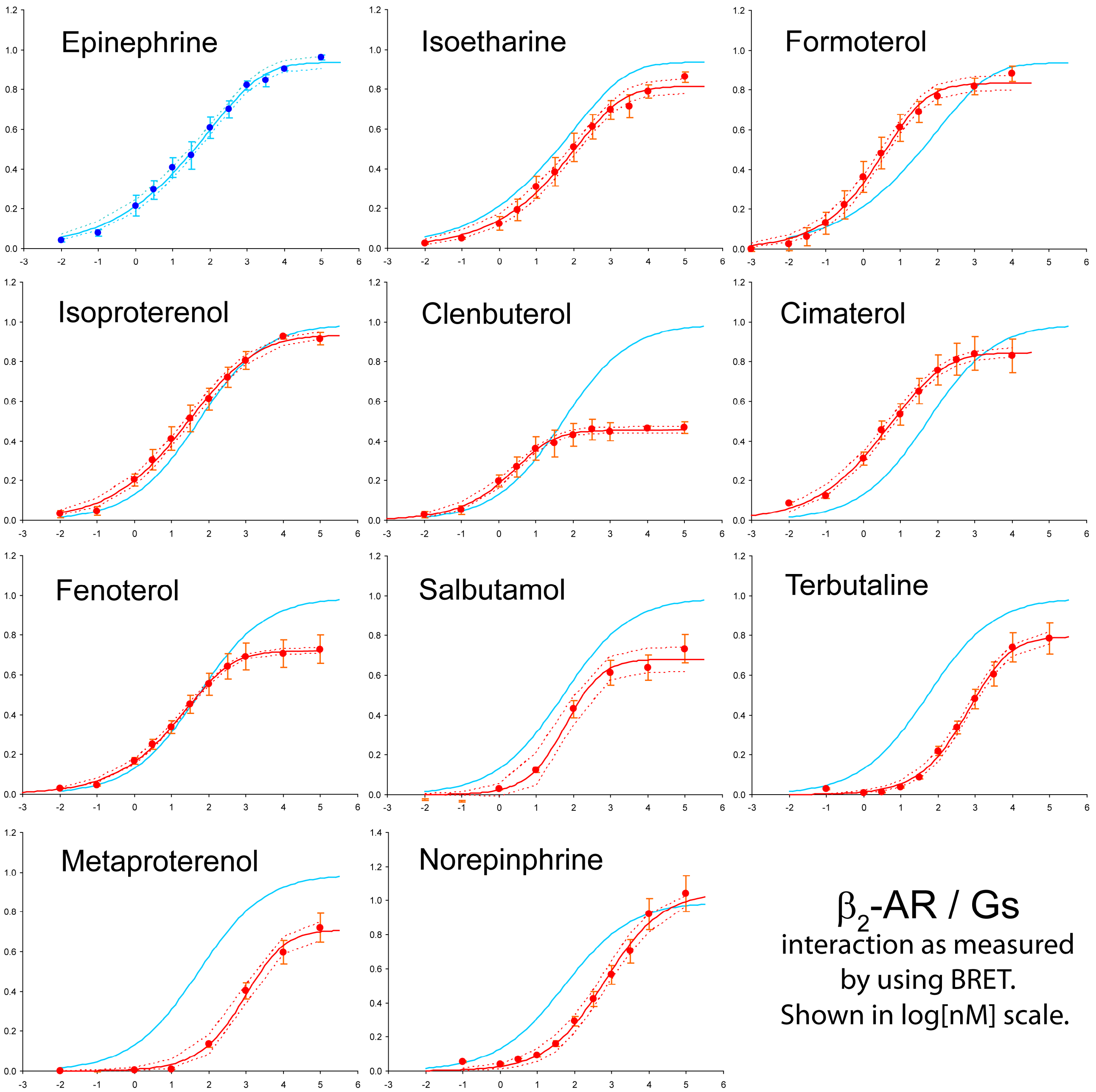

Figure S2. Concentration-Response curves for the reference agonist epinephrine (blue) and test agonists (red) in β_2AR-Gs interactions. Concentration-dependent BRET signals are measured in the membrane preparations from 2B2 cells reconstituted with $G_{\alpha sL}$ and expressing rLuc-β_2AR+rGFP-Gβ_1 that were treated with PTX, as explained in the Methods. All curves are normalized with respect to the maximal response of epinephrine. Solid curves are 3-parameters logistic fits. Dotted curves are the asymptotic 95% confidence band of the regression. Data are mean values from at least four independent experiments based on quadruplicate determinations. Error bars represent standard errors.

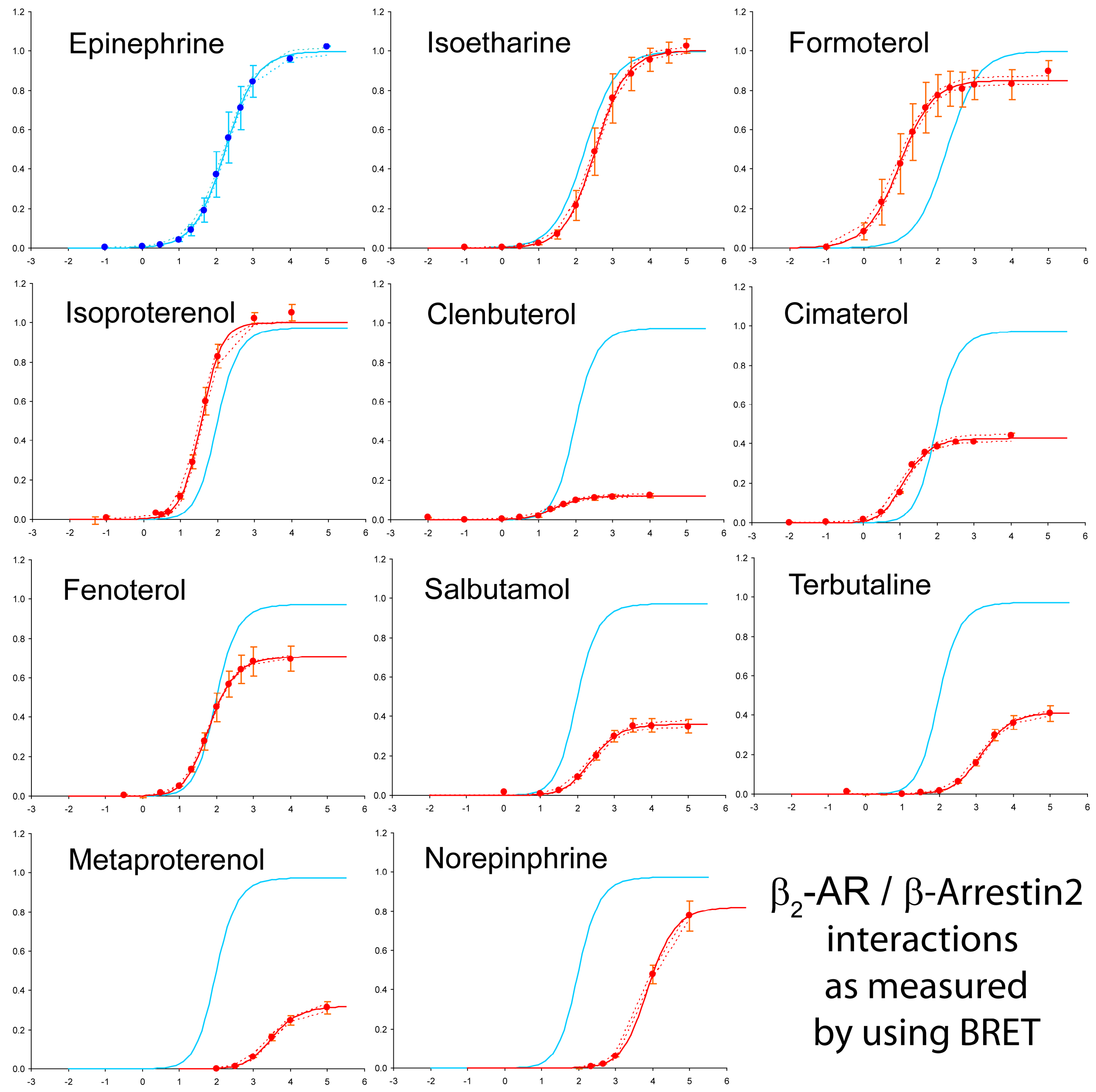

Figure S3. Concentration–Response curves for the reference agonist epinephrine (blue) and test agonists (red) in β_2AR-β arrestin 2 interactions. Concentration-dependent BRET signals were measured in intact 2B2 cell monolayers that do not express any form of Gs, but express rLuc-β_2AR+rGFP-β-Arrestin2 as explained in the Methods. All curves are normalized with respect to the maximal response of epinephrine. Solid curves are 3-parameters logistic fits. Dotted curves are the asymptotic 95% confidence band of the regression. Data are mean values of at least three independent experiments using quadruplicate determinations. Error bars represent standard errors.

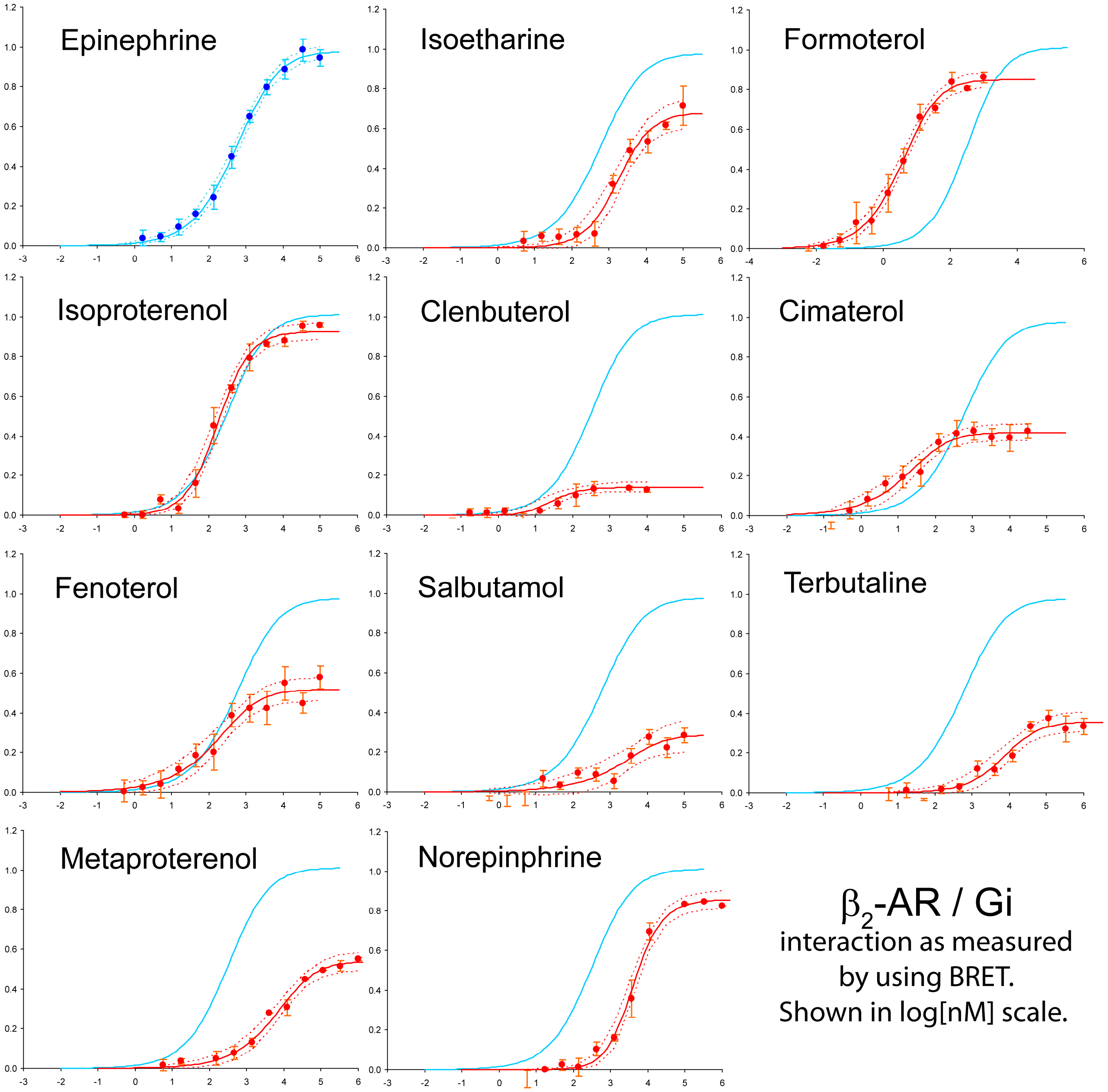

Figure S4. Concentration–Response curves for the reference agonist epinephrine (blue) and test agonists (red) in β_2AR-Gi interactions. Concentration-dependent BRET signals are measured in the membrane preparations from Gs-KO 2B2 cells that express rLuc-β_2AR+rGFP-Gβ_1 in the presence of 10 mM Mg^{++} as explained in the Methods. All curves are normalized with respect to the maximal response of epinephrine. Solid curves are 3-parameters logistic fits. Dotted curves are the asymptotic 95% confidence band of the regression. Data are mean values of at least four independent experiments in quadruplicates. Error bars represent standard errors.

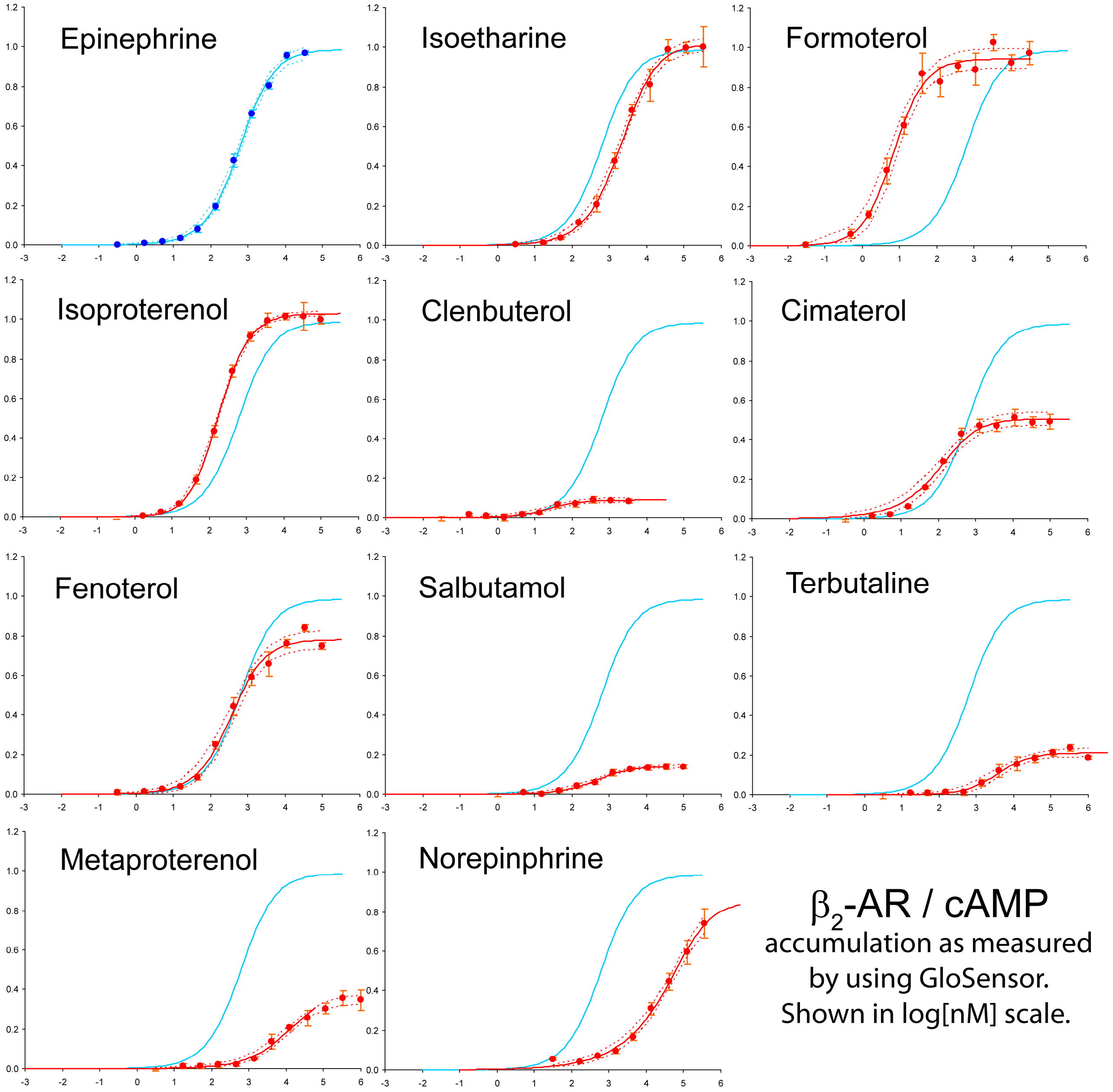

Figure S5. Concentration–Response curves for the reference agonist epinephrine (blue) and test agonists (red) in cAMP assays. Concentration-dependent luminescence signals are measured in HEK-293 cell monolayers that express GloSensor 22F as described in the Methods. All curves are normalized with respect to the maximal response of epinephrine. Solid curves are 3-parameters logistic fits. Dotted curves are the asymptotic 95% confidence band of the regression. Data are mean values of at least three independent experiments in quadruplicate. Error bars represent standard errors.

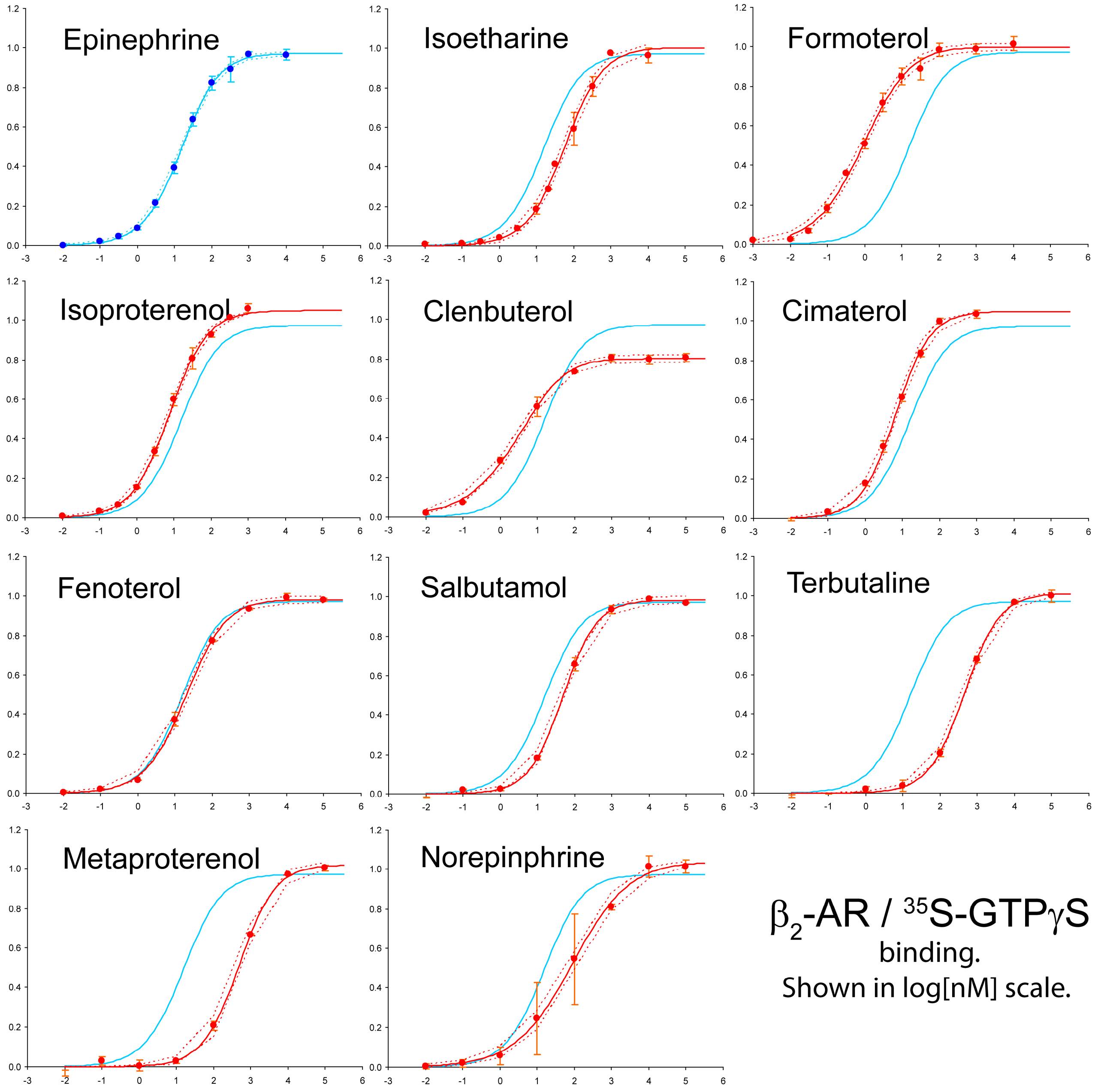

Figure S6. Concentration – Response curves for the reference agonist epinephrine (blue) and indicated test agonists (red) at the indicated assay. Concentration-dependent bindings of GTPγS to Gs were measured in membrane preparations from HEK-293 cells expressing β_2AR-$G_{\alpha sL}$ fusion protein, as described in the Methods. All curves are normalized with respect to the maximal response of epinephrine. Solid curves are 3-parameters logistic fits. Dotted curves are the asymptotic 95% confidence band of the regression. Data are mean values of at least four independent experiments in quadruplicates. Error bars represent standard errors.

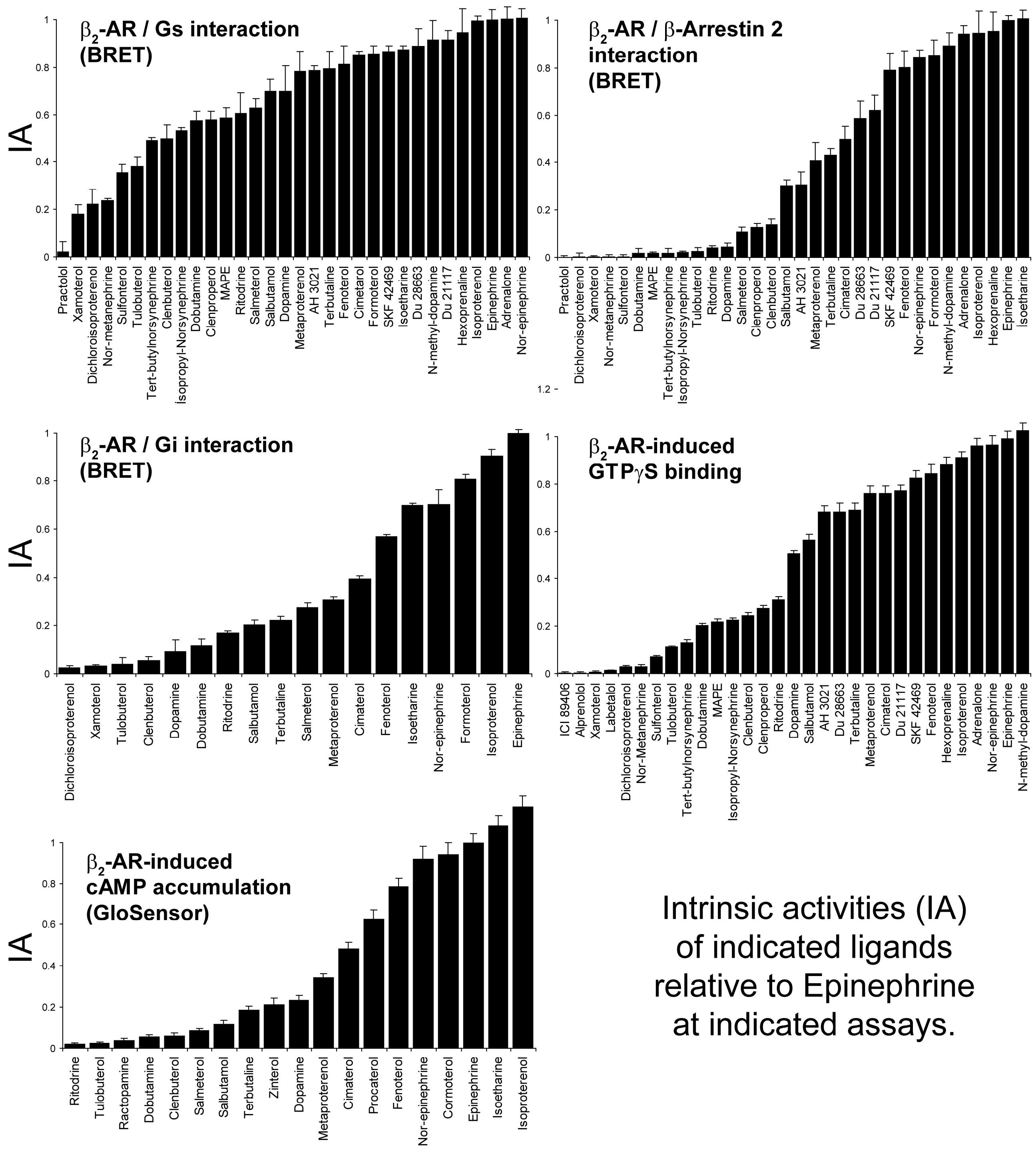

Figure S7. Ligands intrinsic activities in various bioassays. Intrinsic activities (shown as fractional numbers relative to the maximal epinephrine response in each assay) were measured at saturating concentrations of the indicated ligands. β_2AR-Gs or -Gi interactions were measured in cell membranes from 2B2 cells or 2B2 cells reconstituted with Gαs and treated with PTX. β_2AR-arrestin interaction was measured in 2B2 cell monolayers. GTPγS bindings and cAMP accumulations were determined in HEK-293 cells. See Methods for the details. Data are mean values of at least six independent triplicate or quadruplicate experiments. Error bars represent standard errors. These intrinsic activity data constitute the basis of the comparisons given in figures 5 and 6 of the main text.

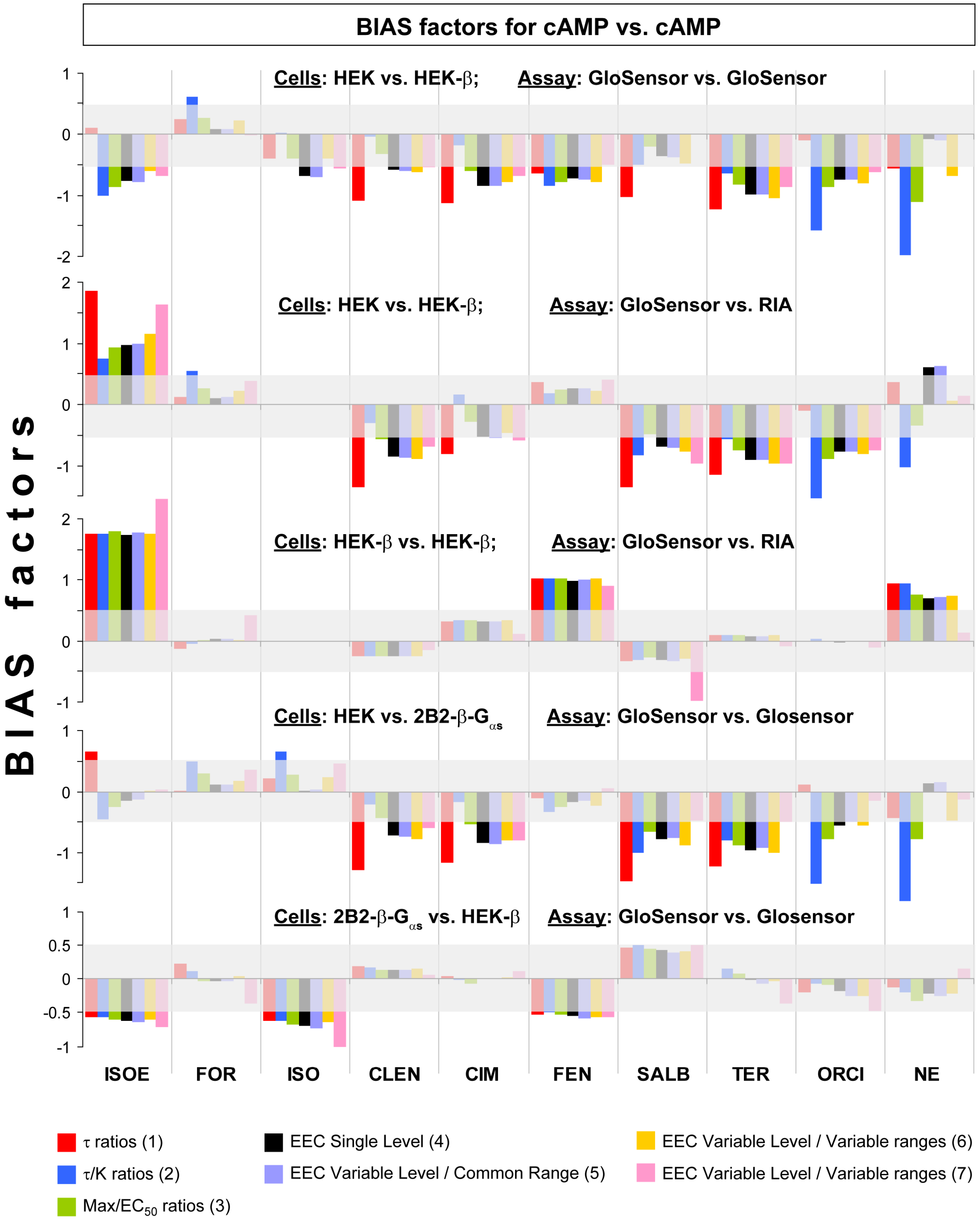

Figure S8. Comparison of cAMP responses observed in different cells and assays. Bias factors were calculated by the indicated methods. Cell names are abbreviated as HEK for HEK-293, HEK-β for HEK 293 cells that overexpress β_2-AR, 2B2-β-$G_{\alpha s}$ for Gs-KO 2B2 cells co-transfected with rLuc-β_2AR+rGFP-$G\beta_1$+$G_{\alpha sL}$. All these cells are also transfected with GloSensor 22F.

Figure S9. Competition binding curves and binding parameters obtained in PTX-treated 2B2-Gs(KO) cell membranes for the indicated ligands.

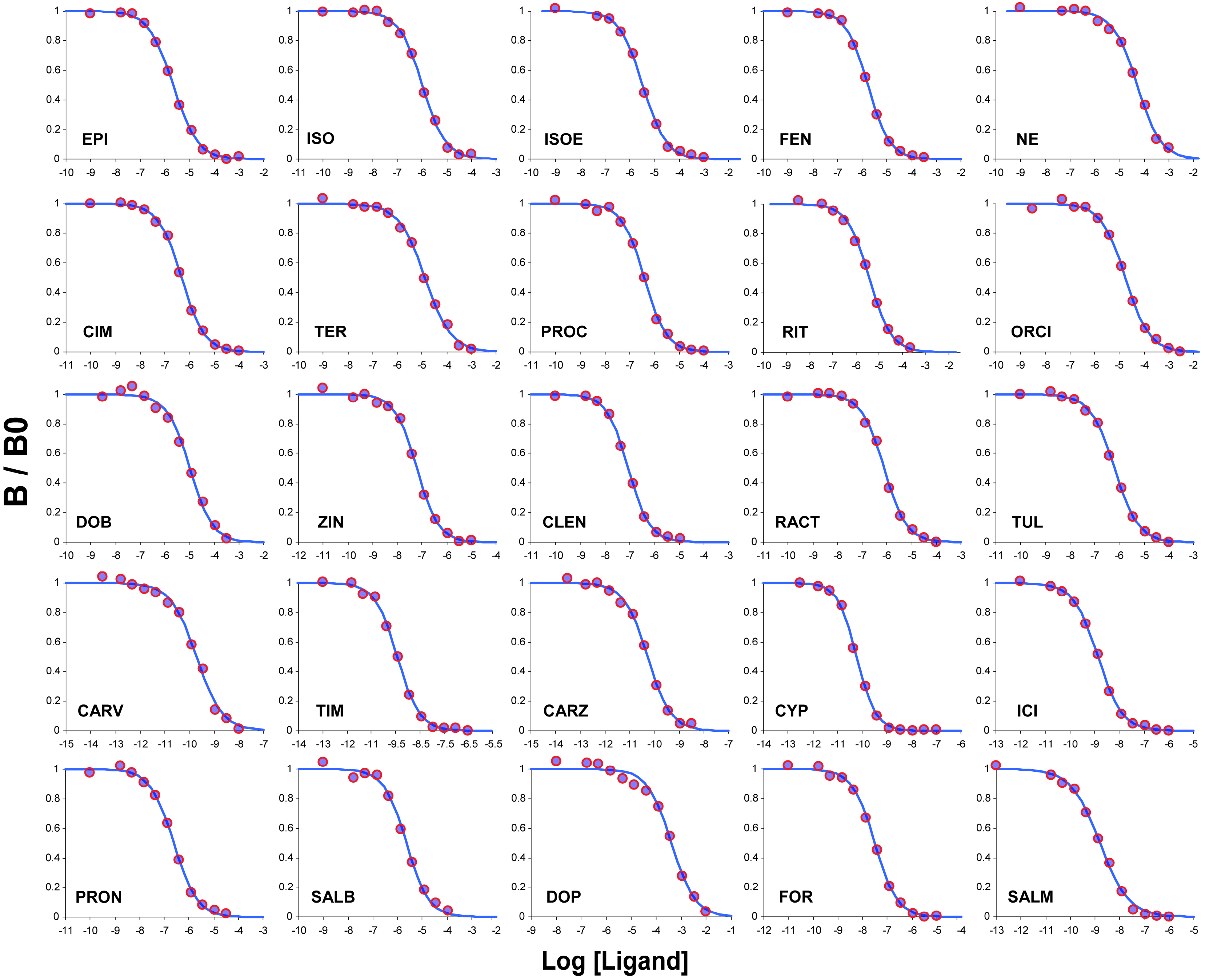

Radioligand : ^{125}I-ICYP
Radioligand Concentration :19 pM
Radioligand Kd : 40 pM
B0 : 6 (+/- 1) pM
***n* = 4 / curve**

Abbrv.	Log(Kd)	SE	Ligand
CARV	-9.9	0.10	Carvedilol
CARZ	-10.5	0.06	Carazolol
CIM	-6.5	0.10	Cimaterol
CLEN	-7.2	0.05	Clenbutarol
CYP	-10.5	0.04	Cyanopindolol
DOB	-5.1	0.10	Dobutamine
DOP	-3.5	0.22	Dopamine
EPI	-5.7	0.16	Epinephrine
FEN	-6.1	0.23	Fenoterol
FOR	-7.6	0.11	Formoterol
ICI118	-9.1	0.04	ICI 118551
ISO	-6.3	0.13	Isoproterenol
ISOE	-5.8	0.04	Isoetharine
NE	-4.4	0.16	Norepinephrine
ORCI	-5.1	0.05	Orcipreinaline
PROC	-6.7	0.09	Procaterol
PRON	-6.8	0.05	Pronethalol
RACT	-6.2	0.20	Ractopamine
RIT	-5.6	0.12	Ritodrine
SALB	-5.9	0.10	Salbutamol
SALM	-8.8	0.16	Salmetarol
TER	-5.0	0.11	Terbutaline
TIM	-9.6	0.04	Timolol
TUL	-6.6	0.23	Tulobuterol
ZIN	-7.1	0.21	Zinterol

Supplementary Methods

Modelling statistical indeterminacy in bias factors computing by Monte Carlo simulations

To evaluate the probability that a mean of bias factors is different from zero, *t*-test statistics is usually applied to error estimates obtained from common uncertainty propagation rules under the assumption of a log-normal distribution. However, such bias factors are not directly measured experimental quantities. They are log differences of parameters estimated from nonlinear fitting of CR curves measured in different bioassays. Thus, the error structure of such determinations is complicated by the diverse fitting routines and model parameter configurations that are used in various methods. In order to evaluate the error structure of bias factors that are estimated by different methods, we used a Monte Carlo strategy.

We simulated theoretical CR curves for 3 ligands, (one reference ligand, Ref, and two test ligands Lig 1, Lig 2), obtained in two different signalling pathways according to the following stimulus-response relationship:

$$r(s) = \frac{r_{\max} s^n}{s^n + K_e^n} \text{ (transfer function), where } s = \frac{\varepsilon[L]}{[L] + K_d}.$$

The 3 agonists have equal efficacy ε in two pathways (unbiased agonists). However the responses have different sensitivities to activated transducers (different K_e and n), so that a significant level of system bias causes divergences in the CR curves of the agonists. The parameters used for simulations are listed in the table below:

	Pathway 1			Pathway 2		
**Parameters**	Ref.	Lig 1	Lig 2	Ref.	Lig 2	Lig 3
System Maximum ($r_{\max}$)	100	100	100	100	100	100
Sensitivity parameter (K_e)	0.01	0.01	0.01	0.1	0.1	0.1
Slope (n)	0.6	0.6	0.6	1	1	1
Binding (K_d)	10	50	1	10	50	1
Efficacy (ε)	10	0.5	0.02	10	0.5	0.02

When applied to the resulting CR curves (figure SM1A, B), six of the seven methods investigated here (see the main text and figure 1 for the labelling of the methods) give zero bias factors for the two test ligands, consistent with the assumptions of the simulation. The only exception is method 3, where not unitary slopes of the CR curves in pathway 1 causes no zero bias. We used these ideal responses to establish the real relationship between error perturbations of the ideal unbiased curves and resulting bias factors calculated by different methods. In fact, small error perturbations in the simulated curves result in deviations from zero bias factors, whose magnitudes depend on both the size of perturbation and type of computation method. Using an automated Matlab script, we generated normally distributed random perturbations of parameters (EC_{50}'s, slopes and maxima of the curves) to generate error that is consistent with the experimental observations (5% coefficient of variation for maxima, fixed standard deviations of 0.1 and 0.05 for log EC_{50}'s and slopes, respectively).

Using the 7 methods under study 200,000 test evaluations were made, each consisting of 6 randomly perturbed curves (3 for each pathway) which were fitted to compute the bias factors for the two test ligands accordingly to the method requirements. The procedure resulted in a total number of 400,000 bias factor values for each method, from which the statistical distributions of bias factors were determined (figure SM1C,D). In case of the full agonist (i.e., Lig 1, in figure SM1C), this analysis shows that random error in CR curve parameters affects the 7 methods almost identically. However, slight differences in distribution widths among the methods (e.g., methods 2 and 7) are noticeable in case of the partial agonist (Lig 2, in figure SM1D). Nevertheless, with the exception of the systematic error introduced by the incorrect usage of method 3 (no unit slopes), the distributions of bias factor estimates are centred at the expected zero bias value. From this analysis we estimate that a bias factor value ≤ 0.5 falls roughly in the 95% band of the distributions even under optimal experimental conditions. Therefore, we considered bias factors in the ± 0.5 range about

zero as insignificant, regardless of their statistical significance assessed by standard rules of error propagation (i.e. from regression parameters of experimentally measured bias factors).

Comparison between Monte Carlo-based and error propagation-based assessments of statistical significance.

To compare statistical decisions based on the Monte Carlo (MC) analysis with those based on *t*-statistics of error-propagated values, we examined a sample of 120 experimentally determined bias factors calculated with the operational model-based methods (i.e. methods # 1 and 2). The data (figure SM1E) were pooled from the experiments shown in figures 3, 4 of the main paper, and represent 10 test agonists analyzed in six different bioassay comparisons (namely, arrestin vs. Gs BRET, Gi vs. Gs BRET, arrestin vs. cAMP-GloSensor, GTPγS binding vs. Gs-BRET, GTPγS binding vs. cAMP-GloSensor, and cAMP-RIA vs. cAMP-Glosensor in low and high receptor expressing cells). The associated error bars were computed by linear propagation of the corresponding asymptotic regression errors of the estimated parameters (i.e. log(τ) or log(τ/K)) obtained by fitting the agonist CR curves with the operational model either as in method 1 or in method 2. As noted in figure SM1E, there was concordance of statistical significance at the 95% confidence level for a majority of data values (77.5%). For the remaining set of data, 25 bias factor values (i.e., 21%) that were found to be statistically significant in *t*-tests analysis did not exceed the 95% confidence interval established by MC, whereas only 2 values (1.5%) were significant in MC but not in *t*-test. This clearly indicates that conventional *t*-statistics has a greater chance of over-estimating the level of significance of experimentally determined bias factors compared to the 95% confidence interval obtained by MC analysis. Therefore, the rate of false positive significant bias values reported in this study would be even greater if the evaluation was based on conventional *t*-statistics.

Estimation of the positions of the observed intrinsic activities on the reference trajectory

The model-free bias analyses presented in the main paper (methods #8 and 9) require the determination of theoretical projections of observed points on the reference trajectory of a reference agonist. The observed points, which are represented by pairs of intrinsic activities (IA) measured at two different responses for given ligands, can deviate from the reference trajectory for two reasons: 1) random measurement errors in the IA's, and 2) systematic errors that can originate from bias, among other systematic factors. We projected the observed points on the reference trajectory under the assumption that the deviations between the observed points and the reference trajectory are due to pure random errors (as in the standard regression assumption). We used these projected (or predicted) points either to determine outlier observations whose deviations cannot be explained by random error (method #9), or to generate rank orders from the nonlinear reference trajectory (method #8). In figure SM2A-C, we schematically explain the procedure of estimating the theoretical points on the trajectory that correspond to the observed points.

Construction of the 95% confidence ellipses of the estimated points on the reference trajectory

Any point on the reference trajectory has an uncertainty in its position that is inherited from the uncertainties in the estimated CR curves of the reference agonist. In method #9, the latter uncertainty needs to be specified to decide whether an observed point is significantly away from its corresponding trajectory point or not. For this purpose, we used the asymptotic 95% confidence bands of the fitted CR curves of the reference agonist to construct confidence ellipses around the trajectory points that are projected from the observed points. This procedure is schematically explained in figure SM2 D-E. Briefly, 95% confidence limits of the x and y coordinates of a given point on the reference trajectory are read off from the confidence limits of the respective image points on the fitted CR curves of the reference ligand. We assumed that errors in different assays are not covariant, as they result from independent measurements. Therefore, axes of the confidence ellipses were oriented parallel to the coordinate axes for both observed and theoretical points.

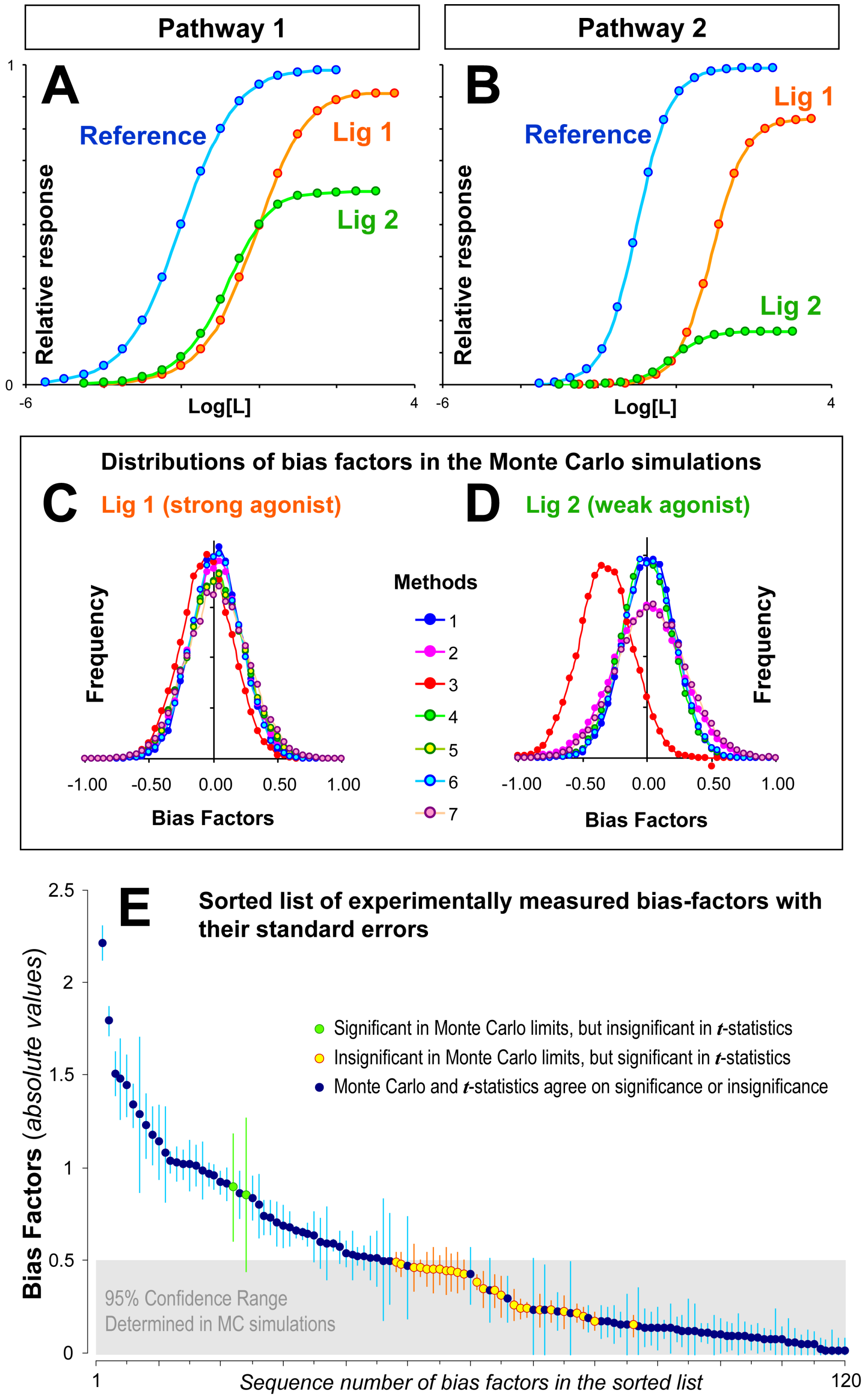

Figure SM1. **Panels A-B** Simulated concentration response-curves of three unbiased agonists (one reference and two tests) in two different pathways are shown (see the text above for simulation details). **Panels C-D**: Distributions of bias factors due to random perturbations in the concentration-response curve parameters are shown. Distributions are given separately for each agonist and method as indicated in the picture. Methods are numbered according to the labels shown in figure 1 of the main text. See the text above for the Monte Carlo simulation details. **Panels E** Absolute values of experimentally determined 120 bias factors that are calculated by means of the operational model (method 1 or 2) are shown in descending order. The confidence region set by the Monte Carlo simulations is shown as a grey shaded area. Bias factors that are: insignificant in the MC limit, but significant in the *t*-test; significant in MC limit, but insignificant in the *t*-test; significant or insignificant both in MC limit and *t*-test are shown with different colours as indicated in the picture.

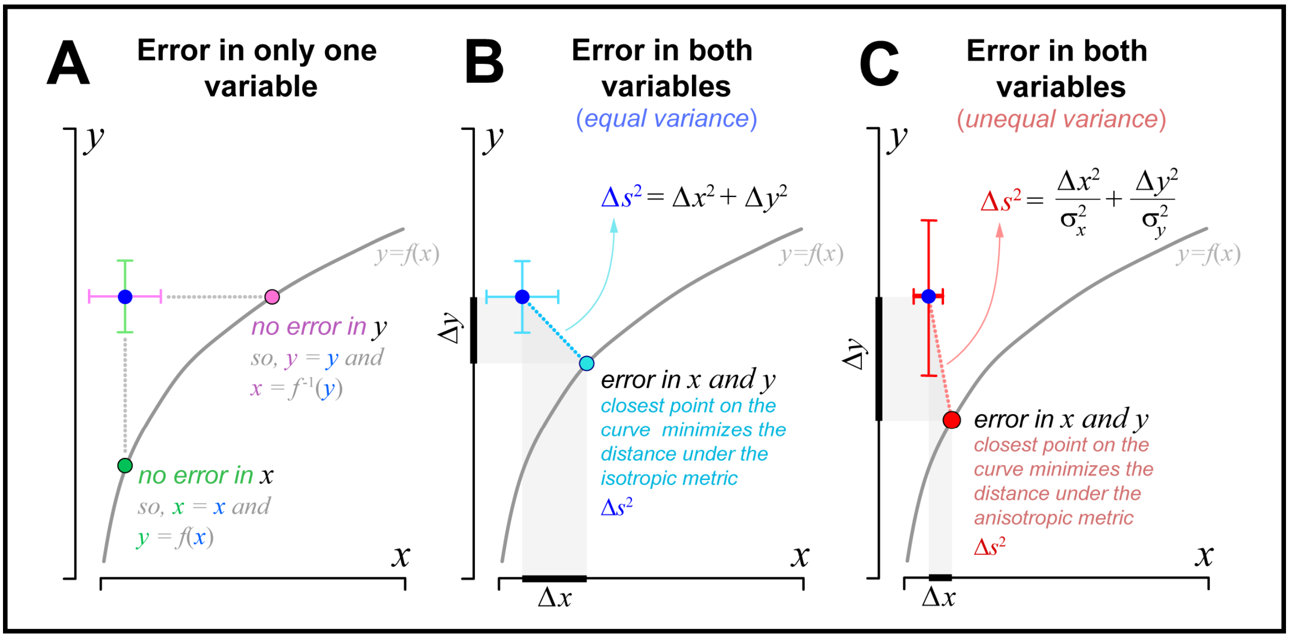

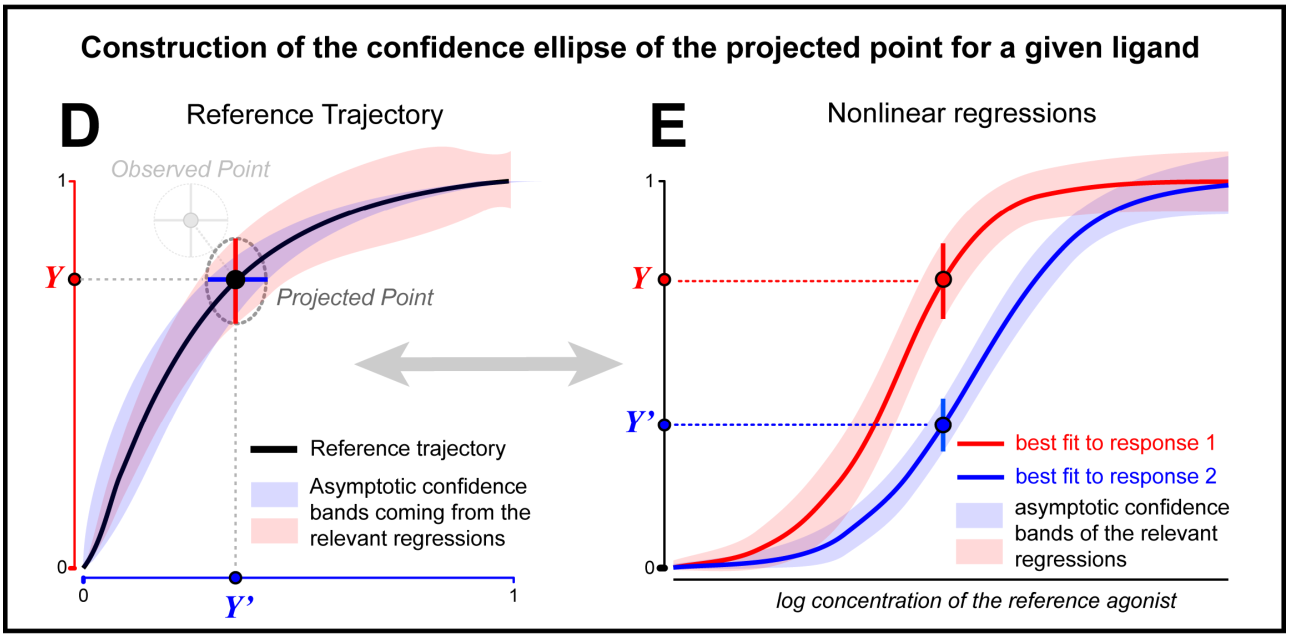

Figure SM2 Panels A-C *Estimation of the "actual" position of a point* [*x,y*] *that is randomly deviated from its theoretical position y = f(x) (i.e. from the reference trajectory).* Three different cases are demonstrated. **Panel A** only one of the variables is assumed to be subject to random error as in the standard regression analysis. In this case, if the x position of the point is known to be error-free, then the projected point on the curve must have the same x as the observed-point, and its y coordinate is given by $y = f(x)$ (green point in **A**). In the reverse case of error-free y, the y coordinates of the observed-point and the projected-point must be the same, but the x coordinate of the projected point is given by $x = f^{-1}(y)$ (magenta point in **A**). In **panel B and C** both variables are assumed to be subject to (independent) random errors. In this case, the best estimate of the projected point can be given, by standard least square arguments, as the trajectory point that minimizes the squared-distance between the curve and the observed point. If the two coordinate-variables x and y have equal variances then the Euclidian (isotropic) metric can be used to measure the relevant distance as shown in **B**. If the two variables have unequal variances (i.e. $\sigma_y^2 \neq \sigma_x^2$), then an anisotropic metric should be more appropriate to measure distances as shown in **C**. In this case, displacements along x and y directions do not contribute equally to the squared distance, but in proportion to the reciprocals of their observed variances. Consequently, the more uncertain is a direction, the less it contributes to the overall distance. In the present analyses, x and y coordinates of an observed point correspond to two intrinsic activities measured in two different responses for a given ligand, whereas the reference trajectory is determined by the CR curves of the reference agonist. Hence, for the observed points $\sigma_y^2 \neq \sigma_x^2$ is expected in general. Therefore, we used the metric given in **C** to measure distances in the minimization procedures. Note that the metric given in **C** is the most general one in the sense that it becomes an isotropic metric when $\sigma_y^2 = \sigma_x^2$ as in **B**, or it can represent the case in **A** when one of the σ's approaches to zero.

Panels D-E *Schematic demonstration of the estimation of the uncertainty of the projected points.* A projected point on the reference trajectory (as in **D**) is subject to a confidence region that is determined by the confidence bands of the regression curves of the reference agonist (as in **E**). Red or blue shaded areas about the reference trajectory (**D**), or about the fitted CR curves (**E**) represent the 95% asymptotic confidence limits of the regression curves as indicated in the picture. It is self evident in the picture that the latter confidence regions imply a confidence ellipse for a given trajectory point. For the obvious reason that the measurements of two responses are strictly independent, no error covariance is assumed between the two responses. Hence the axes of the confidence ellipses are oriented parallel to the coordinate axes. Here, the coordinates of a point is labelled by Y' and Y instead of x and y in order to be consistent with the equation of the reference trajectory given in figure 2A (main paper).
